# Resolving Conformational Preferences of Monosaccharides from ^1^H and ^13^C NMR Chemical Shifts Using an Integrated MD and QM Approach

**DOI:** 10.1021/acs.jcim.6c01310

**Published:** 2026-07-06

**Authors:** Wojciech Plazinski, Göran Widmalm

**Affiliations:** † Jerzy Haber Institute of Catalysis and Surface Chemistry, Polish Academy of Sciences, 30-239 Krakow, Poland; ‡ Department of Chemistry, Arrhenius Laboratory, Stockholm University, S-106 91 Stockholm, Sweden

## Abstract

Solution-state NMR spectroscopy is a powerful experimental technique that provides insight into the molecular structure and dynamics of saccharides in aqueous solution. Computational tools commonly used for modeling carbohydrate conformations, such as molecular dynamics (MD) simulations and quantum mechanical (QM) calculations, provide information on the inherent dynamics of conformational changes and structure-dependent NMR parameters, respectively; however, understanding how NMR parameters depend on dynamic conformational behavior remains challenging. Herein, we present an integrated MD and QM approach to accurately determine NMR parameters, in particular ^1^H and ^13^C NMR chemical shifts of saccharides. Classical MD simulations are used to sample conformational space, and representative structures are subsequently subjected to QM calculations to obtain NMR parameters, which are averaged according to populations in different conformational states. This approach reproduces experimental NMR chemical shifts with high accuracy (MAE = 0.96 ppm for ^13^C and 0.066 ppm for ^1^H relative to experimental data) across 11 monosaccharide entities. In parallel, we establish a quantitative relationship between conformational properties of monosaccharides and the chemical shift values. In this context, empirical relationships between chemical shifts and torsional angles enable mapping of structural descriptors onto NMR observables. Furthermore, we demonstrate that the use of conformation-dependent chemical shifts allows quantitative description of conformational equilibria within monosaccharide molecules. Average chemical shift values assigned to discrete conformers (e.g., ring conformers or hydroxymethyl rotamers) and to atoms in the vicinity of torsion angle transitions are analyzed; populations in distinct conformational states are optimized by minimizing deviations from experimental NMR chemical shifts. This approach enables determination of *gt*:*gg*:*tg* populations of hydroxymethyl group rotamers in β-d-Glc*p*-OMe, α-d-Man*p*-OMe and α-d-Gal*p*-OMe, as well as the chair:inverted chair ratio for the β-d-Ara*p*-OMe six-atom membered ring. Overall, this study establishes a framework in which NMR chemical shifts serve as quantitative probes of carbohydrate conformation, complementing traditional *J* coupling-based analyses.

## Graphical abstract

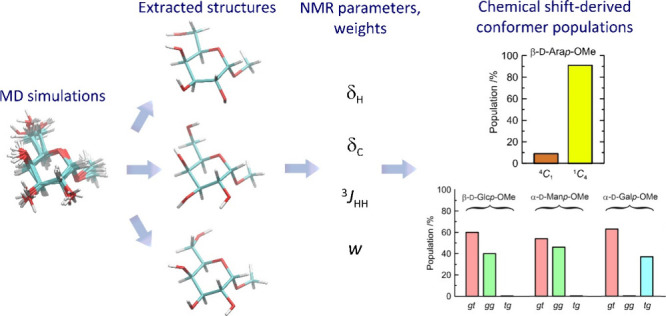

## Introduction

In biology and life sciences carbohydrate-containing molecules are known as glycans and comprise both oligo- and polysaccharides, as well as the conjugated entity of glycoproteins, glycolipids and structures containing an aglycone such as saponins. The biological function of these glycans is vast and includes, inter alia, structural support, pathogen defense, immune evasion, intercellular signaling and adhesion, epigenetic regulation and cell surface interactions.^1^ Monosaccharides are the building blocks of glycans and the common ones are pentoses and hexoses containing five and six carbon atoms, respectively.^2,3^ Naturally occurring monosaccharides can have either the d- or l-absolute configuration^4^ and they can serve as therapeutics for rare diseases,^5^ exert anticancer effects^6^ or be used to produce rare sugars.^7^ Monosaccharide composition and proportion, glycosidic linkage type and anomeric configuration, branching pattern of saccharide chains and molecular weight distributions are factors that influence structural features and the bioactivity of polysaccharides.^8^ Moreover, the number of different monosaccharides is limited to only ten^9^ in the mammalian glycome, which is described as the entirety of glycans and glycoconjugates produced in mammalian cells. By contrast, within the prokaryotic kingdom the number of monosaccharides is considerably larger and for bacteria this difference amounts to almost 2 orders of magnitude.^10^

The cyclic forms of pentoses and hexoses are the furanoid or pyranoid ring forms and exist in equilibria with open aldehyde or hydrate forms for aldoses.^11^ Whereas the conformation of furanoses is described by a puckering amplitude and a pseudorotation phase angle on a pseudorotation wheel relating twist and envelop conformers,^12^ the conformational description of pyranoses includes 38 canonical conformers^13^ described by different forms of chair, half-chair, boat, skew and envelope with three puckering parameters in a spherical polar set, viz., a total puckering amplitude, a polar angle and an azimuthal angle.^14^ Depending on the ring form of these sugars, one or even two additional degrees of freedom are present for exocyclic groups. For hexoses in the pyranoid ring form the exocyclic hydroxymethyl group may populate three staggered conformations resulting in a rotamer distribution at the torsion angle ω, with different relative ratios being dependent on stereochemistry at nearby atoms and substituents on the monosaccharide.

In analysis of conformation and dynamics of carbohydrates in solution, NMR spectroscopy experiments facilitate observation of conformationally dependent parameters such as chemical shift, scalar coupling constants and residual dipolar couplings.^15,16^ In solution the conformational preferences of hexoses in the pyranoid ring form and their exocyclic hydroxymethyl group are often elucidated based on three-bond scalar coupling constants (^3^ *J*_HH_)^17–19^ and NMR residual dipolar coupling constants have been utilized to determine the structure of a methyl pentopyranoside in solution.^20^ Carbon-13 NMR chemical shifts have for a long time been used in the analysis of carbohydrate structure, among other things, due to the large spectral width and often resolved ^13^C singlet resonances in proton-decoupled ^13^C NMR spectra.^21^ The ^1^H NMR chemical shifts of carbohydrates have also been of use in structural analysis of glycans,^22^ though the narrow spectral width in ^1^H NMR spectra as well as the effects of proton–proton coupling constants to the spectra make these chemical shifts more difficult to obtain accurately. More recent developments such as pure shift NMR spectroscopy^23^ thereby resolving the ^1^H resonances of mono- and oligosaccharides into singlets^24,25^ and NMR spin simulations^26^ whereby ^1^H NMR chemical shifts can be obtained of mono- and oligosaccharides^27^ make it possible to obtain this NMR parameter accurately. Classical molecular dynamics (MD) simulations based on empirical molecular mechanics force fields are often employed to elucidate the conformational space accessible to glycans, for example in studies of mono-, di-, and oligosaccharides.^28–32^ The MD simulations have been complemented by a comparison to experimental data from, e.g., small-angle X-ray scattering^33,34^ or NMR spectroscopy.^35–37^ However, even though computational predictions of NMR parameters related to saccharides have been performed^38–42^ the use of NMR chemical shifts in conformational analysis of carbohydrates have to date been limited.

Despite the widespread application of MD simulations and quantum mechanical (QM) calculations in carbohydrate research, a persistent challenge remains in establishing a direct, quantitative link between conformational sampling and NMR chemical shifts. In routine approaches, MD simulations are used to describe conformational equilibria, whereas QM calculations are applied to selected static structures, often without a rigorous framework for integrating these two levels of theory in a statistically consistent manner. As a consequence, the relationship between dynamic conformational behavior and ensemble-averaged NMR observables is frequently treated only qualitatively, limiting the predictive and interpretative power of computational methods.

We herein introduce an MD and QM-based approach to compute conformationally dependent NMR parameters such as ^3^ *J*_HH_, ^1^H and ^13^C NMR chemical shifts. A key aspect of this approach is the systematic selection of representative structures from MD trajectories, enabling efficient yet comprehensive coverage of the conformational space relevant under experimental conditions. A collection of 11 monosaccharide entities for which NMR parameters were determined in this study or form literature are used to develop population-dependent relationships for ^1^H and ^13^C NMR chemical shifts, thereby facilitating additional parameters to be used in conformational studies of carbohydrates with an aim to link derived NMR chemical shifts to molecular conformation.

Importantly, particular emphasis is placed on the sensitivity of both ^13^C and ^1^H NMR chemical shifts to well-defined conformational states of monosaccharides, such as staggered rotamers of the hydroxymethyl group (e.g., *gt*, *gg*, *tg*) of hexopyranosides and distinct ring conformations of pyranosides. This enables the decomposition of ensemble-averaged chemical shifts into contributions arising from discrete conformers, each characterized by a specific structural motif. As a consequence, a unique opportunity arises to exploit experimentally measured NMR chemical shifts — representing ensemble averages — as independent reference data for the reverse determination of conformational populations through optimization within an overdetermined system of relationships in which multiple, independently derived conformer population vs chemical shift dependencies are simultaneously satisfied. In this framework, each conformational state contributes to the overall observed chemical shift according to its population, allowing the experimentally observed values to be decomposed into weighted contributions from discrete structural motifs. By fitting these contributions across a set of chemical shifts that exceed the number of unknown conformer populations, the system becomes overdetermined, enabling statistically robust estimation of desired populations and reducing ambiguity associated with relying on a limited number of NMR observables. A necessary condition for this approach is the ability to define a finite set of discrete conformers that meaningfully represent the underlying conformational landscape. This requirement is commonly fulfilled in the case of saccharides, where well-defined structural states (staggered rotamers of the rotatable exocyclic groups and glycosidic linkages or distinct pyranose ring conformations) can be clearly distinguished and treated as separate contributors to the ensemble. To further improve the averaging over ensembles corresponding to individual, well-defined conformers, we applied methodology developed in our earlier studies,^43^ which is capable of describing conformational^29,43^ and tautomeric^44^ properties of saccharides, as well as determining ensemble-averaged NMR observables.^45,46^

The remainder of this article is organized as follows. First, the experimental and computational protocols (including MD simulations, structure selection, and QM-based NMR calculations) are described in detail. Next, the accuracy of the calculated NMR parameters is evaluated against experimental data for a set of monosaccharides. Subsequently, the relationships between conformational descriptors or discrete sets of conformers and chemical shifts are explored, leading to the formulation of quantitative linkages between structure and NMR observables. In parallel, these relationships are applied to determine conformational equilibria of selected systems, demonstrating the potential of NMR chemical shifts as quantitative probes of carbohydrate conformation.

## Methods

### NMR Experiments and Processing of Data

Samples of monosaccharides and methyl glycosides thereof were prepared in 5 mm outer diameter NMR tubes as solutions in D_2_O. NMR experiments were carried out at 298 K, 310 or 343 K on a 600 MHz Bruker AVANCE III spectrometer equipped with a 5 mm inverse Z-gradient TXI (^1^H/^13^C/^15^N) probe or a 700 MHz Bruker AVANCE III equipped with a 5 mm TCI Z-gradient cryoprobe. ^1^H and ^13^C NMR chemical shifts were referenced to internal 3-trimethylsilyl-(2,2,3,3-^2^H_4_)-propionate (TSP, δ_H_ 0.00) and externally to a 10% solution of 1,4-dioxane in D_2_O (δ_C_ 67.40), respectively. The experimental data were processed and evaluated using Topspin 4.1.4. Refinement of ^1^H NMR chemical shifts and scalar spin–spin coupling constants of β-d-Ara*p*-OMe was based on a QM analysis carried out using a prerelease version of Cosmic Truth (CT; https://ct.nmrsolutions.io), a Web-based, client/server software for automated and semiautomated spectrum analysis from NMR Solutions Ltd. (Kuopio, Finland).

Heteronuclear coupling constants in β-d-Ara*p*-OMe were determined by a one-dimensional long-range (1DLR) NMR experiment^47,48^ at 343 K at 600 and 700 MHz ^1^H frequency using a selective excitation at the resonance frequency of C1 as well as by an IPAP-selHSQMBC^49,50^ experiment at 700 MHz essentially as previously described.^51,52^ A 2D *F*_2_-coupled perfect-^1^H,^13^C-HSQC NMR experiment^53^ was performed on β-d-Ara*p*-OMe at 343 K in D_2_O at a ^1^H frequency of 700 MHz in order to determine the ^1^ *J*_C1,H1_ coupling constant. The FIDs of the 2D experiment were acquired with digital resolution of 0.13 Hz/point in the *F*_2_-dimension and zero-filled once prior to Fourier transformation.

A 1D ^1^H,^1^H-NOESY NMR experiment with a mixing time of 300 ms employing a zero-quantum coherence suppression scheme was performed on β-d-Ara*p*-OMe at 343 K and a ^1^H frequency of 600 MHz using a selective excitation at the resonance frequency of the *O*-methyl group essentially as previously described.^54^

### MD Simulations

The computations concerned single molecules of the 12 monosaccharide entities under investigation, viz., α-l-Fuc*p*-OMe, β-l-Fuc*p*-OMe, α-l-Rha*p*-OMe, β-l-Rha*p*-OMe, α-d-Xyl*p*, β-d-Xyl*p*, β-d-Xyl*p*-OMe, β-d-Ara*p*-OMe (two conformers), β-d-Glc*p*-OMe, α-d-Man*p*-OMe and α-d-Gal*p*-OMe, and involved: (1) MD simulations performed within classical additive force fields and (2) ab initio QM calculations. The chemical formulas of the considered monosaccharides are given in Figure 1 and the nomenclature referring to torsion angles, staggered conformers of hydroxymethyl groups, ring shapes and atom numbering is given in Figure 2. In all cases the ring conformation was assumed to be default for the series of the compound, i.e., the regular ^4^ *C*_1_ and inverted chair ^1^ *C*_4_ for d- and l-series, respectively, with the exception of β-d-Ara*p*-OMe, where both ^4^ *C*_1_ and ^1^ *C*_4_ were considered.

**Figure 1:**
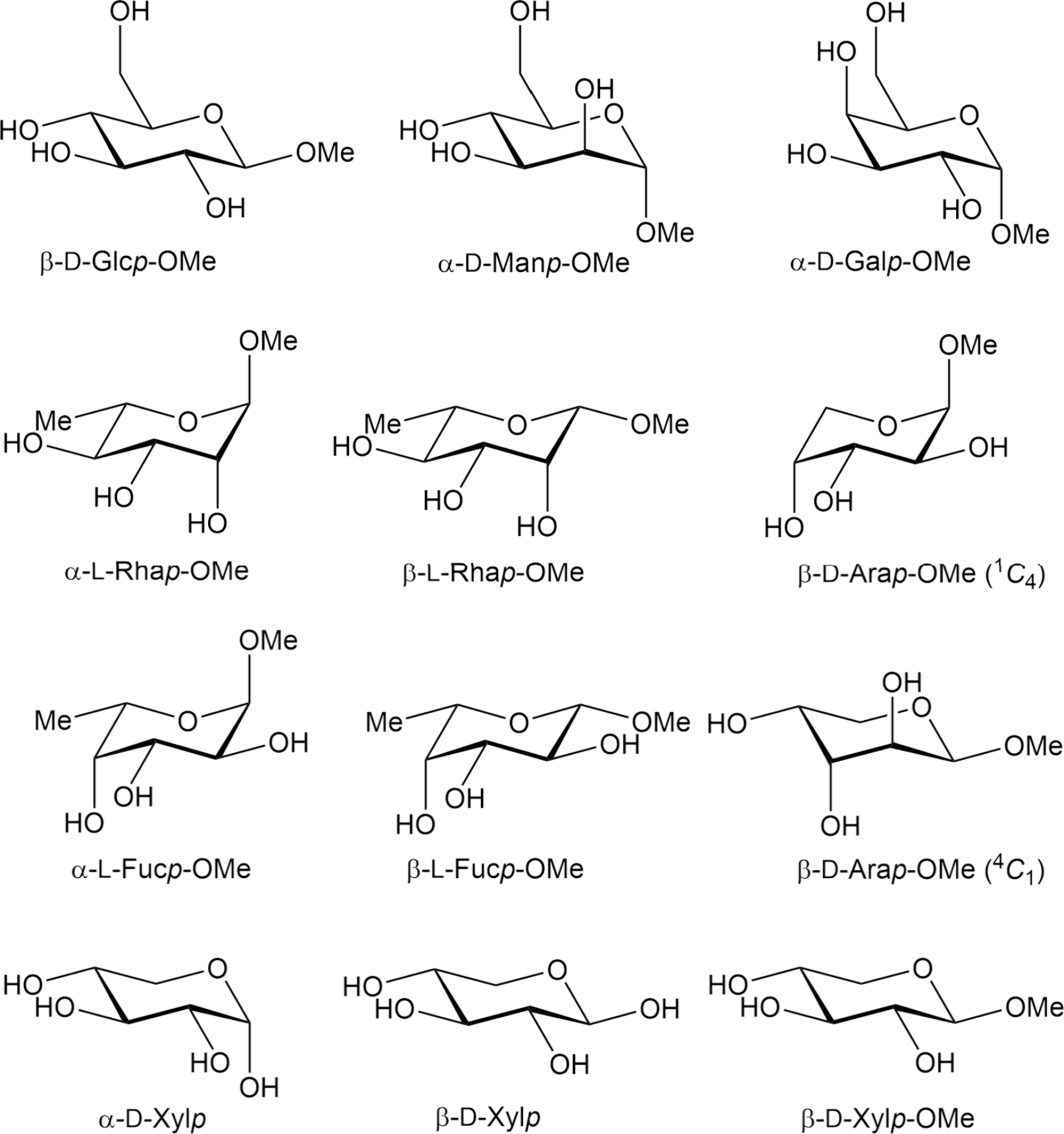
Chemical structures of the monosaccharide entities considered in the present study.

**Figure 2:**
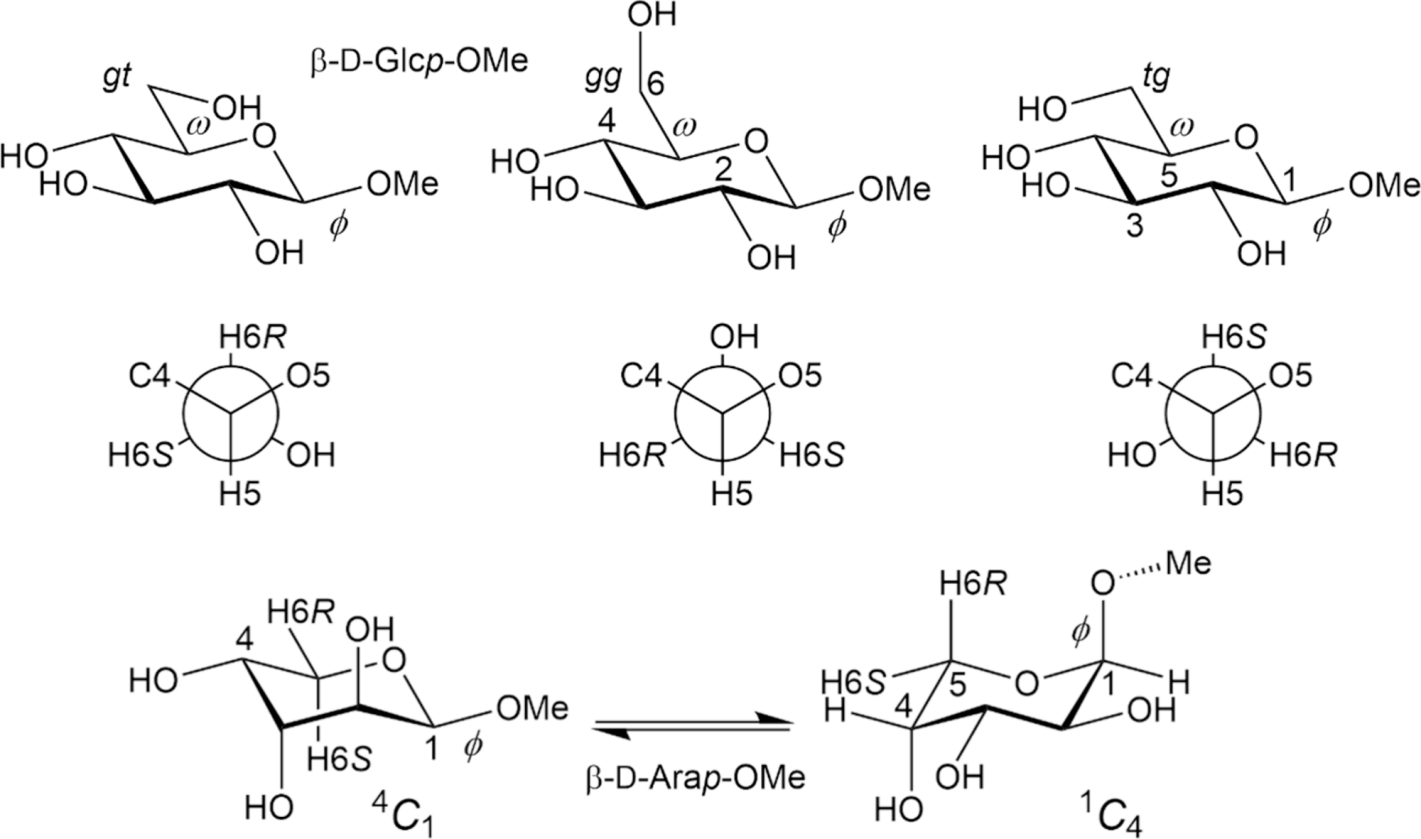
Nomenclature referring to staggered conformers of hydroxymethyl groups (exemplified for β-d-Glc*p*-OMe), ring shapes (exemplified for β-d-Ara*p*-OMe), torsional angles, and atom numbering used in this study. In the ^1^ *C*_4_ chair conformation of β-d-Ara*p*-OMe the *exo*-anomeric conformation at the glycosidic torsion angle ϕ (O5–C1–O1–C_OMe_) and the atoms H1 and H4 have been drawn explicitly (vide infra).

The classical MD simulations were carried out by using the GROMACS 2023.2 package^55^ and the three carbohydrate-dedicated force fields: CHARMM^56^ (all compounds), GROMOS 53a6_CARBO/CARBO_R_ ^57,58^ (β-d-Glc*p*-OMe, α-d-Man*p*-OMe and α-d-Gal*p*-OMe) and GLYCAM06 ^59^ (β-d-Glc*p*-OMe, α-d-Man*p*-OMe and α-d-Gal*p*-OMe). The CHARMM and GLYCAM parameters in the GROMACS-readable format were prepared by using the CHARMM-GUI online server (www.charmm-gui.org)^60^ whereas GROMOS parameters were adopted from our previous work.^58^ The considered MD system consisted of one monosaccharide molecule solvated by ca. 1000 explicit water molecules within a cubic box simulated under periodic boundary conditions. The box edges (of initial dimensions corresponding to ca. 3.2 × 3.2 × 3.2 nm^3^) were preoptimized by a 1 ns constant-pressure MD equilibration at 1 bar and 298 K. After equilibration, the unbiased MD simulation was carried out for 400 ns. The data (energies and trajectory) was saved every 1 ps. During the MD simulation, the temperature was maintained close to its reference value by applying the V-rescale thermostat,^61^ whereas for the constant pressure (1 bar, isotropic coordinate scaling) the Parrinello–Rahman barostat^62^ was used with a relaxation time of 0.4 ps. The equations of motion were integrated with a time step of 2 fs using the leapfrog scheme.^63^ The full rigidity of the water molecules was enforced by application of the SETTLE procedure.^64^ The translational center-of-mass motion was removed every time step separately for the solute and the solvent.

Certain details in the simulation setup varied according to the applied force field.

CHARMM: The TIP3P model of water^65^ was applied. The hydrogen-containing solute bond lengths were constrained by application of the LINCS procedure with a relative geometric tolerance of 10^–4^.^66^ The electrostatic interactions were modeled by using the PME method^67^ with cutoff set to 1.2 nm, while van der Waals interactions (LJ potentials) were switched off between 1.0 and 1.2 nm.

GROMOS: The SPC model of water^68^ was applied. The solute bond lengths were constrained by application of the LINCS procedure with a relative geometric tolerance of 10^–4^.^66^ The nonbonded interactions were calculated using a single cutoff distance set to 1.4 nm and a Verlet list scheme. The reaction-field correction was applied to account for the mean effect of the electrostatic interactions beyond the long-range cutoff distance, using a relative dielectric permittivity of 61.

GLYCAM: The TIP3P model of water^65^ was applied. The hydrogen-containing solute bond lengths were constrained by application of the LINCS procedure with a relative geometric tolerance of 10^–4^.^66^ The electrostatic interactions were modeled by using the PME method^67^ with the cutoff set to 1.0 nm, while van der Waals interactions (LJ potentials) were switched off between 1.0 and 1.1 nm.

For each system, simulated within each considered force fields, various set of temperatures was applied, varying from 298 to 343 K. This was motivated by the desire to reflect the conditions that correspond to the experimental data discussed in the paper. Independently of the MD simulations of the saccharides, a simulation was carried out on a single molecule of dioxane in water, with the setup corresponding to the CHARMM force field and a simulation length of 100 ns.

The analysis of the unbiased MD simulations included mainly the conformation of the hydroxymethyl group expressed by the value of the O5–C5–C6–O6 torsion angle (ω). This conformation was assigned to one of the three possible staggered conformers, based on the ω value, i.e., *gt* (staggered conformation at 60°), *gg* (−60°), and *tg* (180°). Additional analyses involved the conformation of all rotatable hydroxyl of methoxyl groups present in a given monosaccharide; this was done by considering the distribution of the values of the C_*n*+1_-C_*n*_-O_*n*_-H_*n*_ or O5–C1–O1-C_OMe_ torsional angles, respectively.

### Metadynamics Simulations

The metadynamics-based free energy calculations were restricted to the following monosaccharide entities: β-d-Ara*p*-OMe, α-d-Xyl*p*, β-d-Xyl*p* and β-d-Xyl*p*-OMe, all simulated within CHARMM force field. Calculations were focused on the 1D free energy profile associated with the distortion of the pyranose ring. The Cremer-Pople θ parameter^14^ was selected as the metadynamics coordinate (collective variable^69^). Simulations were performed by using the PLUMED 2.2 software^70^ and relied on the well-tempered metadynamics protocol^71,72^ using Gaussian local functions of widths 2.86°, an initial deposition rate of 0.01 kJ·mol^–1^·ps^–1^ and a parameter Δ*T* of 1788 K (see eq 2 in the study by Barducci et al.^72^). The duration of metadynamics simulations was 50 ns and the convergence was checked by calculating ring-inversion free energy values as a function of time. The remaining details of the simulation setup were identical to those described earlier for the case of unbiased MD simulations.

### Selection of the Data for QM Calculations

One of the main purposes of the MD simulations was to provide the set of molecular configurations of monosaccharides to be used as an input data for the QM calculations. At this stage, only the simulations carried out within the CHARMM force field were taken into account. The selection of configurations passed to QM calculations was done in the two alternative ways.

1. The procedure described in our previous works^43,46^ was applied. First, the conformation of all rotatable bonds associated with exocyclic hydroxyl, methoxyl and hydroxymethyl groups of the molecule was analyzed to identify all main rotamer types and to determine the quantitative definitions of these rotamers. Second, the population of each individual conformation of the whole molecule, differing only by the combined types of all rotamers, was calculated. Third, a single, representative conformer was selected for each of the individual conformations mentioned above. This resulted in a number of extracted structures varied from 16 to 150, depending on the system. This procedure was applied to all compounds considered. Subsequent RMSD analyses of the QM-optimized configurations confirmed that the individual conformational identities were retained, without changes in the assigned rotamer types.
2. The selection was made on the basis of the ω torsion angle value. For each bin of the 2-degree width, a single frame from an MD trajectory was extracted. This resulted in extracting ca. 180 structure per system. This procedure was applied only in the case of monosaccharide entities that contain a hydroxymethyl moiety, i.e., β-d-Glc*p*-OMe, α-d-Man*p*-OMe and α-d-Gal*p*-OMe.

Regarding the structures collected according to the first method, the values of the NMR parameters, i.e., ^1^H (δ_H_) and ^13^C (δ_C_) NMR chemical shifts as well as the ^3^ *J*_HH_, ^3^ *J*_CH,_^2^ *J*_HH_ and ^1^ *J*_CH_ coupling constants, were calculated by using the following expression for a weighted average:

$$X=\frac{\overset{N}{\underset{i}{\sum }}p_{i}X_{i}}{\overset{N}{\underset{i}{\sum }}p_{i}}$$ 1

where X = ^3^ *J*_HH_, ^3^ *J*_CH,_^2^ *J*_HH_,^1^ *J*_CH_, δ_C_ or δ_H_, *p*_*i*_ is the population of the *i*th conformer, according to the MD simulations, *X*_*i*_ is the corresponding *X* parameter value and *N* is the number of considered structures. In the cases where we considered the values of δ_C_ and δ_H_ corresponding to a subset of configurations, representing, e.g., only *gt*, *gg* or *tg* rotamers of a hydroxymethyl group, the input data (i.e., the type of configurations and *N*) were modified accordingly. For structures collected according to the second method, averaging was not performed.

### QM Calculations and Recovering the NMR Parameters

The QM calculations were performed in Gaussian09 ^73^ at the DFT/ωB97XD/6-31+G­(d,p) level of theory^74,75^ and in the presence of implicit, aqueous solvent (Polarizable Continuum Model model).^76^ The geometry optimization was performed by using the default criteria, except for the use of the *tight* keyword, tightening the cut-offs on forces and step size. Subsequently, spectroscopic parameters were calculated for the fully optimized structures using the GIAO (gauge-independent atomic orbital) approach^77^ at the same level of theory. The *mixed* keyword was invoked to request a two-step spin–spin coupling calculation.^78^

For configurations collected by applying the first approach constraints were not applied on any degree of freedom. For configurations extracted using the second method, in order to determine the dependence of the chemical shift values on a broad range of the ω values, the QM-based optimization and calculation of the NMR parameters relied on applying the constrained ω value with respect to their initial, MD-derived, values.

The values of chemical shifts (δ_i_) were calculated from isotropic nuclear magnetic shielding constants (σ) by assuming a linear relation:^79^

$$\delta_{i}=\sigma_{\mathrm{ref}}-\sigma_{i}$$ 2

where σ_ref_ is a reference value. The value of σ_ref_ was treated as adjustable parameter, determined by assuming minimal deviation of the experimental from the theoretical δ values. Usually, the common value of σ_ref_ was determined for the whole set of considered compounds, separately for δ_C_ and δ_H_. However, in the case of compounds containing a hydroxymethyl moiety (β-d-Glc*p*-OMe, α-d-Man*p*-OMe and α-d-Gal*p*-OMe) the σ_ref_ values were adjusted for a subset of experimental data (involving only the chemical shifts of atoms in the vicinity of rotating group, i.e., δ_C4_, δ_C5_, δ_C6_, δ_H5_, δ_H6*R*_, δ_H6*S*_) in order to minimize the deviations and facilitate further data analysis (see the next subsection).

Additionally, we calculated the value of σ_ref_ independently, using the reference compound, i.e., dioxane. In this case, the σ_ref_ value was calculated as a nonweighted average over σ_i_ values, obtained by using 40 random configurations extracted from the corresponding MD trajectory and applying the QM calculation scheme described above. At this stage, the analysis was restricted to comparing the calculated value with σ_ref_ determined independently by using eq 2 and the experimental data. The ^3^ *J*_HH_ scalar couplings for β-d-Ara*p*-OMe were calculated as described by Altona^80^ for vicinal coupling constants using eq 11 therein with pertinent coefficients for substituent parameters utilizing the MestReJ 1.1 software.^81^

^3^ *J*_CH_, ^1^ *J*_CH_ and ^2^ *J*_HH_ coupling constants were considered only for the C1,H5_pro*‑S*_, C1,H1 and H5_pro*‑R*_,H5_pro*‑S*_ atom pairs, respectively, and two ring conformers of β-d-Ara*p*-OMe. ^3^ *J*_CH_ and ^2^ *J*_HH_ coupling constants were calculated by using the previously described QM setup and averaged by eq 1. As shown in the study by Jessen et al.,^82^ the accuracy of QM methods in predicting ^1^ *J*_CH_ values may deteriorate when explicit solvent effects are additionally included, especially in the case of hybrid-exchange functionals. Therefore, we repeated the calculations using several alternative QM setups to determine the best agreement with the experimental data, in particular with the measured ^1^ *J*_C1H1_ value as well with the expected gap of ca. 10 Hz between values computed for ^1^ *C*_4_ and ^4^ *C*_1_ ring conformers. The following combinations of functionals and basis sets were applied: BLYP/6-31+G­(d,p)^75,83,84^ (both PCM water^76^ and vacuum), B3LYP/6-311G++(d,p)^75,83,84^ (PCM water), B3LYP/aug-cc-pVTZ^83–85^ (PCM water), and B3LYP/EPR-III^83,84,86^ (both PCM water and vacuum). The best performance was obtained using the BLYP/6-31+G­(d,p) level of theory, in the absence of an implicit solvent model. This QM setup was then used to calculate the ^1^ *J*_CH_ values using the GIAO method, based on the previously optimized conformations of two ring conformers of β-d-Ara*p*-OMe. The final values were obtained by averaging according to eq 1.

### Other Details of the Data Analysis

The dependencies of the theoretically calculated chemical shifts δ_C6_, δ_H6*R*_ and δ_H6*S*_ on the value of the torsional angle ω were expressed by the following empirical equation:

$$\delta \left(\omega \right)=\alpha_{0}+\overset{4}{\underset{i=1}{\sum }}\alpha_{i}\cos\left(i\omega +\beta_{i}\right)$$ 3

where α_i_ and β_i_ are adjustable parameters of values depending on the considered system and atom type. The α_0_ value is dependent on and proportional to the adjusted value of σ_ref_ (eq 2).

The populations of rotamers of hydroxymethyl groups relied solely on the values of δ_C4_, δ_C5_, δ_C6_, δ_H5_, δ_H6*R*_ and δ_H6*S*_ and were calculated by minimizing value of the following expression:

$$\Delta =\underset{j=\left\{\delta \right\}}{\sum }\frac{\left|\Delta_{j}\right|}{\delta_{j,\mathrm{expt}}}$$ 4

where {**δ**} is a set of considered types of atoms and NMR chemical shifts corresponding to them; this set includes δ_C4_, δ_C5_, δ_C6_, δ_H5_, δ_H6*R*_ and δ_H6*S*_ or arbitrarily selected subsets. δ_*j*,expt_ is the experimental value of a chemical shift of a given, *j*th atom and Δ_*j*_ is defined as follows:

$$\Delta_{j}=100\delta_{j,\mathrm{expt}}-\left(\delta_{j,gt}p_{gt}+\delta_{j,gg}p_{gg}+\delta_{j,tg}p_{tg}\right)$$ 5

In eq 5 δ_*j*,*gt*_, δ_*j*,*gg*_ and δ_*j*,*tg*_ are conformation-dependent chemical shifts, corresponding to the given rotamer of hydroxymethyl group (known from theoretical calculations) and *p*_*gt*_, *p*_*gg*_ and *p*_*tg*_ are the optimized populations of these rotamers expressed in [%].

The populations of the regular (^4^ *C*_1_) and inverted chair (^1^ *C*_4_) conformers of β-d-Ara*p*-OMe relying solely on the complete set of chemical shift values were calculated by minimizing value of the expression identical to eq 4 but with {**δ**} defined now as the complete set of experimentally available chemical shifts and Δ_*j*_ is defined as follows:

$$\Delta_{j}=100\delta_{j,\mathrm{expt}}-\left(\delta_{j,4C1}p_{4C1}+\delta_{j,1C4}p_{1C4}\right)$$ 6

In eq 6 δ_*j*,4C1_ and δ_*j*,1C4_ are conformation-dependent chemical shifts, corresponding to the *j*th atom and given shape of pyranose ring (known from theoretical calculations) and *p*_4C1_ and *p*_1C4_ are the optimized populations of the ring conformers expressed in percent [%]. Additionally, the analogous calculations relying on eqs 4 and 6 were carried out using other conformation-dependent NMR parameters instead of the chemical shifts appearing in eq 6, such as the full set of ^3^ *J*_HH_ coupling constants or individual values of ^1^ *J*_CH_ and ^2^ *J*_HH_ coupling constants.

Eqs 4–6 are crucial for determining conformational equilibria in saccharide molecules. They are based on knowledge of the set of chemical shift values corresponding to a finite number of well-defined conformers; such information can be theoretically determined using the methods employed in this study.

Additionally, experimental data on chemical shifts for atoms located in the region where conformational changes occur are necessary. Since the number of such atoms (and consequently, the size of the set {**δ**} in eq 4 is usually greater than the number of considered conformers, the relationships describing the average values of all sets of δ_C_ and δ_H_ concerning the number and populations of conformers form an overdetermined set of equations. Therefore, instead of solving such a system of equations — which would result in a mathematical inconsistency — the approach proposed in the current work is based on minimizing deviations from the experimental data using eqs 4–6.

## Results and Discussion

### Experimental NMR Spectra

^1^H and ^13^C NMR chemical shift assignments of β-d-Ara*p*-OMe were carried out using experiments suitable for carbohydrates^87,88^ and from the ^1^H NMR spectrum at 700 MHz (Figure S1) the chemical shifts as well as the ^*n*^ *J*_HH_ coupling constants were refined by NMR spin-simulation (Table 1). The quantum mechanics-driven ^1^H iterative functionalized spin analysis (QM-HiFSA) approach^26^ facilitates detailed analysis of complex scalar coupling networks. Notably, a five-bond proton–proton coupling constant across the pyranoid ring was possible to identify and quantify being ∼0.6 Hz between H1 and H4, in particular by comparing the multiplet of the H4 proton (Figure 3a), the excellent agreement for the spin-simulated peak in the scalar coupled network (Figure 3b), and the deviation between the ^1^H resonance in the experimental spectrum and the one where the ^5^ *J*_H1,H4_ coupling had been removed in the simulation (Figure 3c).

**Table 1:** ^1^H and ^13^C NMR Chemical Shifts (ppm) of β-d-Ara*p*-OMe at 343 K in D_2_O Referenced to TSP (δ_H_ 0.00) and Dioxane in D_2_O (δ_C_ 67.40)^a^ ^,^ ^b^

compound		1	2	3	4	5	5	OMe
β-d-Ara*p*-OMe	δ_H_	4.825	3.847	3.831	3.996	3.858^*R*^	3.664^*S*^	3.419
	^3^ *J*_*n*,*n*+1_	3.80	10.02	3.57	1.55^*R*^; 2.31^*S*^			
	δ_C_	100.75	69.13	69.74	69.67	63.34		56.10

a ^1^H NMR chemical shifts and ^*n*^ *J*_HH_ (Hz) were refined by NMR spin-simulation (Cosmic Truth).

b Additional scalar coupling constants (Hz): ^2^ *J*_H5pro‑*R*,H5pro‑*S*_ −12.78, ^4^ *J*_H1,H3_ −0.43, ^5^ *J*_H1,H4_ 0.58, ^4^ *J*_H1,H5*R*_ −0.61, ^4^ *J*_H1,H5*S*_ –0.12, ^4^ *J*_H2,H4_ –0.43.

**Figure 3:**
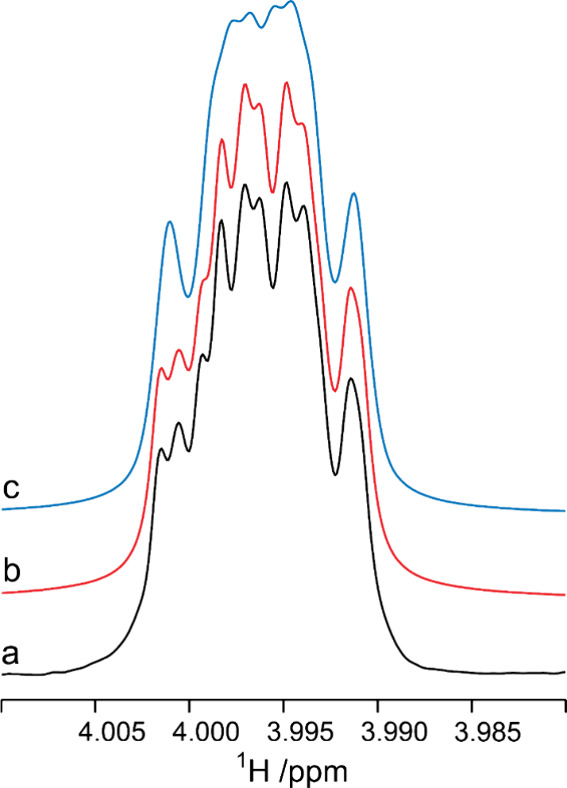
H4 resonance from (a) the ^1^H NMR spectrum at 700 MHz of β-d-Ara*p*-OMe in D_2_O at 343 K (black), (b) the corresponding one from a simulated ^1^H NMR spectrum by total-line shape analysis using the Cosmic Truth software (red), and (c) with the ^5^ *J*_H1,H4_ scalar coupling taken out in the NMR spin-simulation procedure (blue).

The conformational preference of the pyranoid ring of β-d-Ara*p*-OMe was assessed by three-bond homo- and heteronuclear scalar coupling constants as well as by ^1^H,^1^H-NOEs. A ^1^H,^13^C-selHSQMBC-IPAP NMR experiment with selective excitation at H5_pro‑*S*_ (δ_H_ 3.66) resulted in ^3^ *J*_C1,H5pro‑*S*_ of 7.6 Hz and ^1^H-detected 1DLR NMR experiments with selective excitation at C1 (δ_C_ 100.75) showed three-bond correlations to the *O*-methyl group (δ_H_ 3.42) and to H5_pro‑*S*_ (δ_H_ 3.66). The latter experiment was carried out at two magnetic fields; at a ^1^H 600 MHz frequency ^3^ *J*_C1,OMe_ = 4.4 Hz and ^3^ *J*_C1,H5pro‑*S*_ = 7.5 Hz and at 700 MHz ^3^ *J*_C1,OMe_ = 4.4 Hz and ^3^ *J*_C1,H5pro‑*S*_ = 7.6 Hz, detected as antiphase peak-separation and extracted by a *J*-doubling procedure for the more complex peak-pattern, as exemplified for H5_pro‑*S*_ at 700 MHz (Figure S2). Furthermore, the relatively large ^1^ *J*_C1,H1_ coupling constant of 169.8 Hz indicated an equatorially oriented C1–H1 bond.^88,89^

A 1D ^1^H,^1^H-NOESY NMR experiment with selective excitation at the resonance frequency of the *O*-methyl group resulted in a strong NOE to H1 and an NOE of medium intensity to H5_pro‑*R*_ (δ_H_ 3.86) consistent with the *exo*-anomeric conformation^90^ at the glycosidic torsion angle ϕ and the ^1^ *C*_4_ chair conformation of the pyranoid sugar. Moreover, as a reference point for a subsequent comparison we have chosen the β-linked methyl glycoside of the Lewis^a^ trisaccharide (Le^a^). The ^1^H NMR chemical shift for H5 of the α-l-Fuc*p* residue in Le^a^ is predicted by the CASPER program^91^ to be 4.76 ppm, i.e., a large downfield chemical shift displacement of 0.74 ppm has taken place in the trisaccharide relative to that of α-l-Fuc*p*-OMe (δ_H5_ 4.02),^92^ which has the ^1^ *C*_4_ chair conformation as deduced from calculated NMR chemical shifts (vide infra). In a β-linked 8-(methoxycarbonyl)­octyl glycoside of a Lewis^a^ trisaccharide analog^93^ that instead has a β-d-Ara*p* residue (^2^ *J*_H5pro‑*R*,H5pro‑*S*_ – 12.5 Hz), which is the 5-demethyl derivative of α-l-Fuc*p*, the corresponding chemical shift displacement for H5 (axially oriented) was 0.88 ppm. Thus, not only are the chemical shift displacements of the H5 atoms in the two Lewis^a^ trisaccharides similar in magnitude, but the ^2^ *J*_H5pro‑*R*,H5pro‑*S*_ coupling constants are closely similar in β-d-Ara*p* of the Lewis^a^ analog and in β-d-Ara*p*-OMe, which indicates that the β-d-Ara*p* residue has the ^1^ *C*_4_ conformation as the main one in the Lewis^a^ analog, like for β-d-Ara*p*-OMe.

### General Remarks on the Calculated NMR Chemical Shifts

The ^1^H and ^13^C NMR chemical shift values for the complete set of C and H atoms, compared with the corresponding experimental data, are illustrated in Figure 4 (chemical shifts for C) and Figure 5 (chemical shifts for H). The value of σ_ref_, adjusted to minimize the deviation of the magnetic shielding constants from the experimental chemical shift values (eq 2), is equal to 196.196 and 31.727 ppm for C and H atoms, respectively. These values were selected based on the full data set and are shared across all considered compounds.

**Figure 4:**
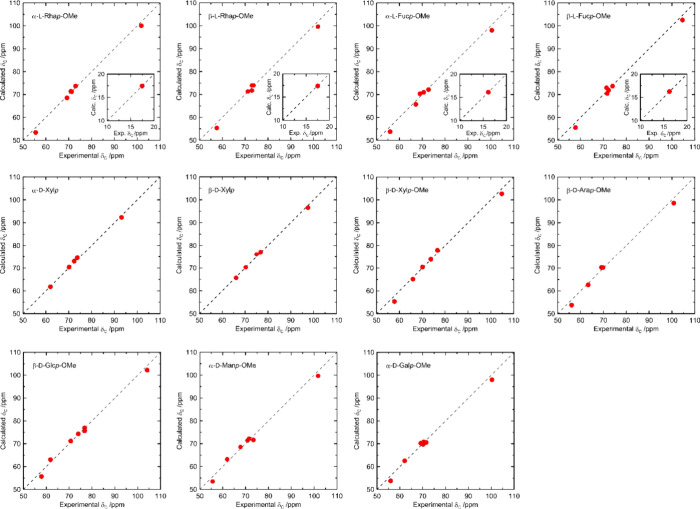
Values of NMR chemical shifts δ_C_ calculated for all considered monosaccharides and averaged by using eq 1, compared to the experimental data. The theoretical data correspond to the QM calculations using the MD-extracted structures and carried out at the DFT/ωB97XD/6-31+G­(d,p) level of theory. The data for β-d-Ara*p*-OMe correspond to the inverted ^1^ *C*_4_ chair conformer of the ring.

**Figure 5:**
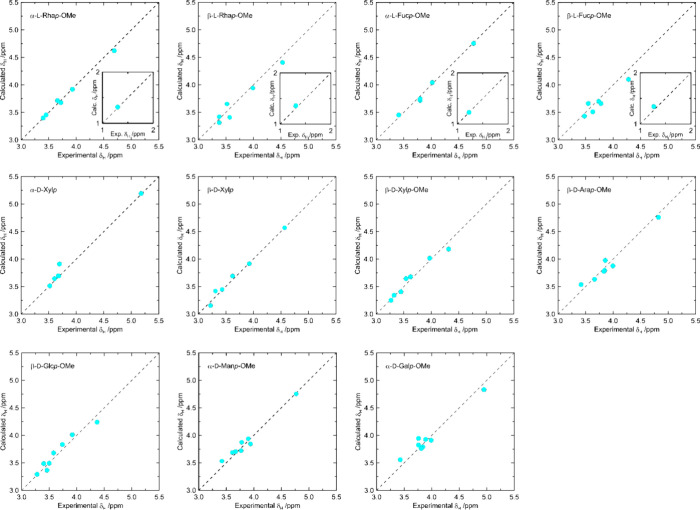
Values of NMR chemical shifts δ_H_ calculated for all considered monosaccharides and averaged by using eq 1, compared to the experimental data. Other details are in Figure 4.

Analogous σ_ref_ values, determined a priori based on QM calculations for dioxane (used as a reference compound for all experimental data), are 197.591 and 28.030 ppm, respectively. Despite the relatively similar values, the deviations are large enough — especially for the chemical shifts of hydrogen atoms — to justify the procedure of selecting σ_ref_ based solely on its relation to experimental data.

It is important to note that the σ_ref_ value itself does not affect the correlation between theoretical and experimental data but merely determines the shift of theoretical data along the *y*-axis (calculated data). However, this value does influence the absolute differences between calculated and experimental chemical shifts by proportionally scaling the mean deviation between these two data sets.

The mean absolute error (MAE) of theoretical data compared to experimental ones for the entire data set is 0.96 and 0.066 ppm for C and H atoms, respectively (Table S1). Relative to the respective maximal values of chemical shift variability, these values correspond to approximately 1.1 and 1.3%, which indicates very good agreement between theory and experiment. Analysis of Figures 4 and 5, as well as Table S1, which contains MAE values for individual compounds, shows that the degree of agreement with experiment is similar for each studied compound (MAE varies within the range of 0.52–1.22 ppm for C and 0.028–0.098 ppm for H). Furthermore, relatively larger deviations for δ_C_ values for a given compound are often compensated by smaller deviations in δ_H_ values (and vice versa).

In the above analysis, for β-d-Ara*p*-OMe, only the chemical shift values determined for the ^1^ *C*_4_ ring conformer were considered. The same values calculated for the ^4^ *C*_1_ conformation correspond to significantly higher MAE values (Table S1), which will be discussed further in a section of the article (vide infra).

Regarding alternative methods for determining NMR chemical shifts of saccharides based on QM calculations, it is worth noting that the average error in the present calculation is of a similar magnitude compared to the results presented by Palivec et al.^42^ (MAE for H atoms: 0.067 ppm vs 0.066 ppm herein; MAE for C atoms: 1.11 ppm vs 0.96 ppm herein). This is despite the fact that we used a single fitting parameter, σ_ref_ (whereas in the study by Palivec et al.^42^ two parameters were optimized) and that our QM calculations involved a smaller number of structures per compound. However, both studies highlight the importance of accounting for a larger number of molecular conformations, which allows for obtaining results averaged over the conformational space explored by saccharides under near-ambient conditions (i.e., temperature close to 298 K and aqueous solution).

Figure 6 presents the same theoretical vs experimental chemical shift values but separated by topologically analogous atom types. This type of comparison allows for checking whether the inaccuracies in the determined δ_C_ and δ_H_ values are random across different molecular fragments or if they are mainly localized in specific regions of the molecule. The corresponding MAE values are provided in Table S2.

**Figure 6:**
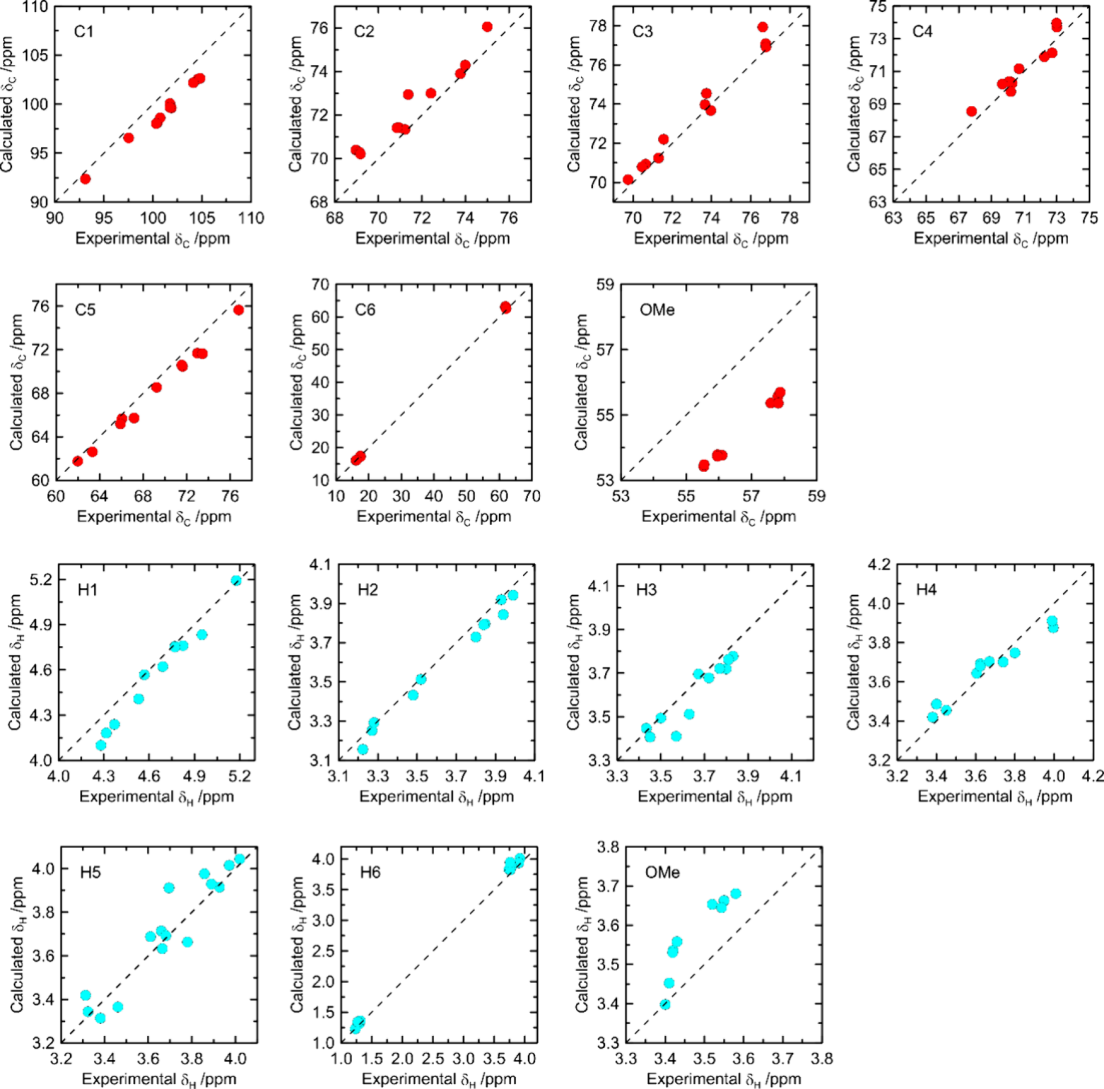
Values of NMR chemical shifts δ_C_ and δ_H_ calculated for all considered monosaccharides are arranged in a way that shows the trend of data variability for topologically analogous atoms in the molecules. The data are the same as those shown in Figures 4 and 5. Independently, the data retrieved using a different methodology regarding only atoms C4, C5, C6, H5, H6*R*, and H6*S* in molecules of β-d-Glc*p*-OMe, α-d-Man*p*-OMe and α-d-Gal*p*-OMe monosaccharides are shown in Figure 10.

The largest deviations correspond to C and H atoms within the methoxy group. These deviations are predominantly systematic, with predicted δ_C_ values being underestimated by an average of 2.22 ppm and δ_H_ values being overestimated by an average of 0.077 ppm. Although we do not have a clear interpretation of the cause of this effect, it can be speculated that it originates from the spatial solvent-exposed positioning of the *O*-methyl group relative to other parts of the molecule. This could lead to a greater (and systematic) influence of inaccuracies associated with the use of the implicit solvent model. The second-largest, though less systematic, MAE values correspond to the C1 and H1 atoms, which are the closest to the *O*-methyl group.

### Conformation of the Pyranose Ring in Pentoses and Methyl Glycosides Thereof

Due to the significant discrepancy observed between the calculated chemical shifts for the ^4^ *C*_1_ conformation of β-d-Ara*p*-OMe and the experimental data, as well as the satisfactory agreement for the alternative ^1^ *C*_4_ ring conformation, one can speculate about the dominance of the latter conformer under experimental conditions. Furthermore, this observation suggests that δ_C_ and δ_H_ data may be used for qualitative and potentially quantitative estimation of trends in the conformational equilibrium of saccharide molecules.

To estimate the populations of the ^4^ *C*_1_ and ^1^ *C*_4_ conformers based solely on chemical shift values, eq 6 was used to minimize the deviations of theoretical data (expressed as a weighted average) relative to the experimental data. The deviations, as defined in eq 4, were scaled according to the magnitude of each chemical shift value to avoid overestimating the weights of higher chemical shifts. The conformation-dependent chemical shift values for the ^1^ *C*_4_ conformer are illustrated in Figures 4 and 5 in the respective panels, while analogous data for the ^4^ *C*_1_ conformer are shown in Figure S3. The optimization of conformer populations was performed for the entire data set but separately for δ_C_ and δ_H_. In both cases, very similar ^4^ *C*_1_:^1^ *C*_4_ population ratios were obtained: 9:91 (for optimization based on δ_C_ values) and 8:92 (for optimization based on δ_H_ values).

Independently of the ring conformer population estimation, ^3^ *J*_HH_ coupling constants (Table 1, vide supra) were calculated using a Haasnoot-Altona equation^80^ and MD trajectories generated during CHARMM force field simulations. A graphical representation of the results is shown in Figure 7. In this case, the disagreement between theoretical and experimental data for the ^4^ *C*_1_ conformer is even more striking than in the case of chemical shifts; the corresponding MAE values are 0.51 Hz for the ^1^ *C*_4_ conformation and 3.77 Hz for the ^4^ *C*_1_ conformation (Table S3). Population optimization using ^3^ *J*_HH_ values and eqs 4 and 6 (where δ values were replaced with ^3^ *J*_HH_ values) leads to a ^4^ *C*_1_:^1^ *C*_4_ population ratio of 0:100, meaning that assuming any nonzero population of the ^4^ *C*_1_ conformer only worsens the agreement between theoretical data and the experiment.

**Figure 7:**
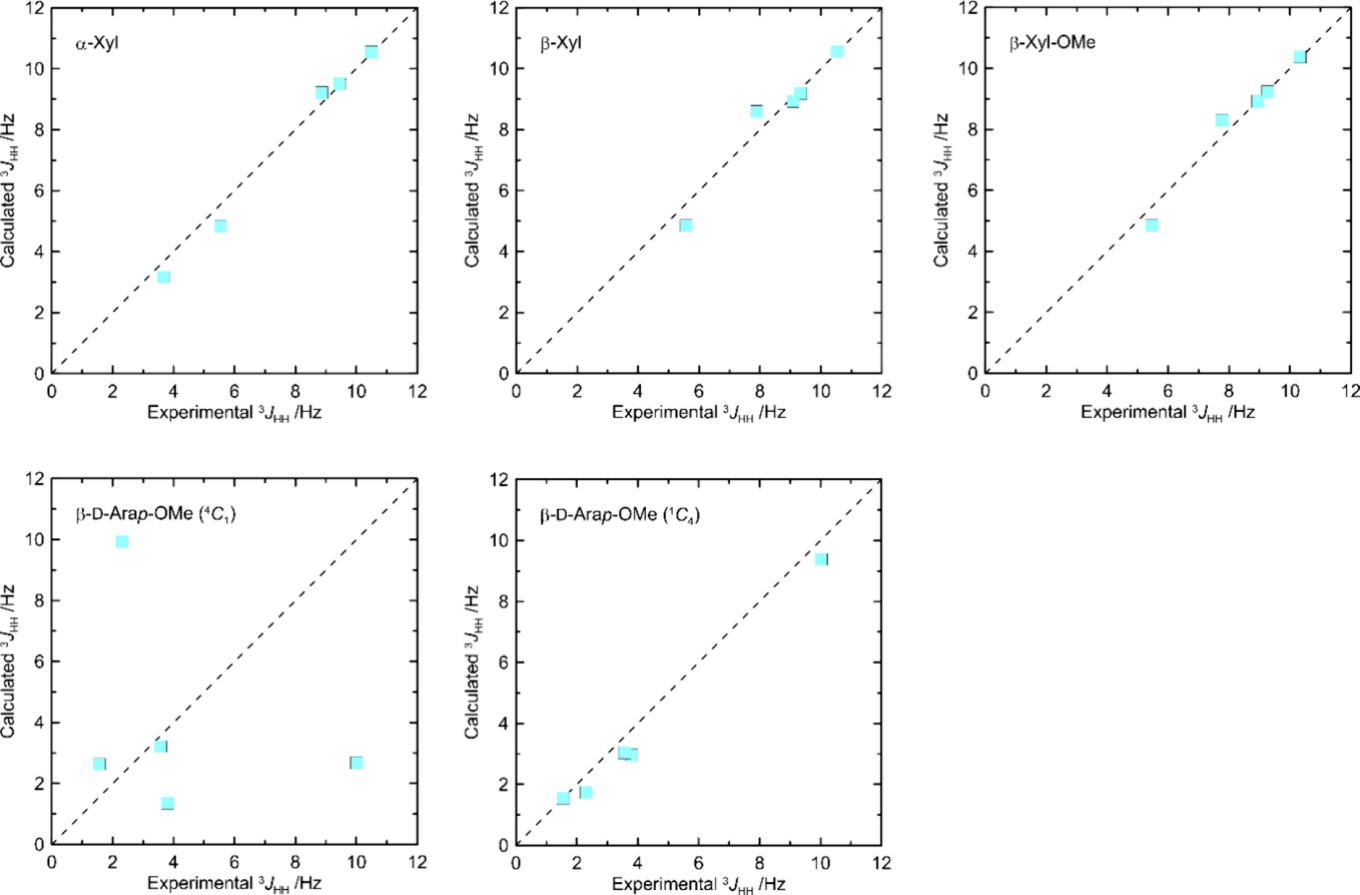
Calculated vs experimental ^3^ *J*_HH_ presented as absolute values for α-d-Xyl*p*, β-d-Xyl*p* and β-d-Xyl*p*-OMe and the two ring conformers of β-d-Ara*p*-OMe. Calculations relied on the Haasnoot-Altona equation and unbiased MD simulations within the CHARMM force field.

A high population of the ^1^ *C*_4_ conformer is further confirmed by the analysis of the ^3^ *J*_CH_ coupling constant values between atoms C1 and H5_pro‑*S*_. From different NMR experiments, this value is 7.5–7.6 Hz (vide supra). According to QM calculations, the average values of this quantity, obtained using eq 1, are 7.73 or 2.53 Hz for the β-d-Ara*p*-OMe ring, which is either in the ^1^ *C*_4_ or ^4^ *C*_1_ conformation, respectively. Furthermore, based on eq 6, where δ values were replaced with ^3^ *J*_CH_ values, and a ^3^ *J*_CH_ value of 7.55 Hz from experiments the conformer populations of the pyranoid ring yielded a ratio of ^4^ *C*_1_:^1^ *C*_4_ = 3.5:96.5. In addition, the QM-computed geminal ^2^ *J*_H5pro‑*R*, H5pro‑*R*_ coupling constant for the geometry optimized β-d-Ara*p*-OMe in the ^1^ *C*_4_ conformation was −13.0 Hz whereas for the ^4^ *C*_1_ conformation it was −11.4 Hz, which for the former is consistent with the experimentally determined two-bond coupling constant of −12.8 Hz (Table 1). Optimization carried out using eqs 4 and 6 with these values of ^2^ *J*_HH_ yielded a chair conformer ratio of ^4^ *C*_1_:^1^ *C*_4_ = 12.5:87.5. Furthermore, the QM-computed ^1^ *J*_C1,H1_ coupling constant in the ^1^ *C*_4_ conformation was 171.4 Hz whereas for the ^4^ *C*_1_ conformation it was 161.3 Hz; the former value is in a good agreement with the experimentally determined value of 169.8 Hz. The larger coupling constant computed for the ^1^ *C*_4_ conformation further supports its predominance as the main chair conformation of this pentopyranoside monosaccharide. The optimization performed by using eqs 4 and 6 yielded a conformer population ratio of ^4^ *C*_1_:^1^ *C*_4_ = 16:84. It may be noted that potential strong coupling effects in *F*_2_-coupled ^1^H,^13^C-HSQC NMR spectra related to an anomeric C–H pair, leading to deviations from the true value of a ^1^ *J*_CH_ coupling constant compared to the apparent ^1^ *J*_C1,H1_ coupling constant measured, are anticipated to be small.^94^

Moreover, ring conformer populations were estimated using metadynamics simulations within the CHARMM force field, resulting in a ^4^ *C*_1_:^1^ *C*_4_ ratio of 4:96, which is close to the values estimated from chemical shift data. This is equivalent to the free energy change associated with the ^4^ *C*_1_ → ^1^ *C*_4_ rearrangement, which is −9.2 kJ/mol. The free energy change associated with the transition from ^4^ *C*_1_ to boat/skew-boat (*B*/*S*) conformers is 16.3 kJ/mol, which justifies considering only chair conformers in the analysis based on eq 6.

In the context of arabinopyranose ring conformations, available crystallographic data from the PDB database (1ABE, 1BAP, 1MMZ, 3TB6, 4NZF, 4QDP, 5LA2, and 6ABP) primarily concern β-l-Ara*p* and indicate that this residue adopts the ^4^ *C*_1_ ring conformation (10 out of 21 structures) and a twisted boat conformation (11 out of 21 structures). For the five PDB entries with reported real-space correlation coefficient (RSCC) values (1MMZ, 3TB6, 4NZF, 4QDP, and 5LA2), all arabinopyranose residues exhibited RSCC values between 0.85 and 1.00, corresponding to at least an acceptable fit to the electron density, with six of these structures displaying a very good fit according to the criteria of Guvench and Straffin.^95^ Given that β-l-Ara*p* and β-d-Ara*p* are mirror images, this corresponds to a preference of β-d-Ara*p* for the ^1^ *C*_4_ rather to the ^4^ *C*_1_ ring shape. Notably, values obtained from alternative theoretical methods also indicate a high degree of flexibility for the β-d-Ara*p* ring. The semiempirical scheme by Angyal^96^ estimates a ^4^ *C*_1_:^1^ *C*_4_ ratio of 30:70 for β-d-Ara*p*, whereas simulations of the same compound using the GROMOS 53a6_CARBO_R_ force field^28^ predict a ^4^ *C*_1_:^1^ *C*_4_ ratio of 75:25, suggesting — contrary to the present data — a predominance of the ^4^ *C*_1_ conformation.

The ring conformation of α-d-Xyl*p*, β-d-Xyl*p* and β-d-Xyl*p*-OMe was also investigated relying ^3^ *J*_HH_ coupling constants, obtained in conjunction with ^1^H and ^13^C NMR chemical shift assignments,^97^ within the pyranoid ring (Table S4, Figure 7). In the case of xylopyranoses, the agreement between experimental and theoretical ^3^ *J*_HH_ values is markedly improved compared to β-d-Ara*p*-OMe. As shown in Table S3, the mean absolute errors for α-d-Xyl*p*, β-d-Xyl*p* and β-d-Xyl*p*-OMe are 0.34, 0.35, and 0.26 Hz, respectively, i.e., consistently lower compared to the value obtained for β-d-Ara*p*-OMe being in the ^1^ *C*_4_ conformation (0.51 Hz). Inspection of the individual coupling constants (Table S4) reveals that the theoretical predictions reproduce the experimental data with high fidelity across all vicinal proton pairs, with only minor deviations observed, particularly for the *J* couplings of the H1,H2 and H4,H5_pro‑*R*_ pairs. The uniformly low MAE values and the absence of large discrepancies analogous to those observed for the ^4^ *C*_1_ conformer of β-d-Ara*p*-OMe indicate that, for the investigated xylopyranoses, the dominant ring conformation is well described by the ^4^ *C*_1_ geometry without the need to invoke alternative shapes. This is in line with the ring-distortion free energies determined for the remaining monosaccharide entities, i.e., α-d-Xyl*p*, β-d-Xyl*p*, and β-d-Xyl*p*-OMe, which confirm their strong preference for adopting the ^4^ *C*_1_ conformation. The corresponding energies associated with the ^4^ *C*_1_ → ^1^ *C*_4_ transitions range from 10.9 to 16.4 kJ/mol, with the lowest value corresponding to α-d-Xyl*p*. The analogous quantities for the ^4^ *C*_1_ → *BS* rearrangements vary from 14.2 to 23.1 kJ/mol. These results justify considering a single chair conformation for xylopyranoses at the stage of selecting the conformation for the QM calculations, as described in the Methods section.

Detailed numerical values of ring-distortion free energies are given in Table S5 in the Supporting Information. It is worth noting that the free energy changes obtained in this study for xylopyranoses are in close agreement with the data reported by Guvench et al.,^98^ where the same compounds were investigated under similar conditions. The small differences, ranging from 0.6 to 2 kJ/mol, arise from differences in enhanced sampling simulation protocols as well as the chosen coordinates defining the ring conformation.

### Conformation of the Hydroxymethyl Group in Methyl Hexopyranosides

#### Initial Remarks

The results described in the previous subsection confirm that chemical shifts, analogous to other spectroscopic parameters (in particular, *J* coupling constants), can be used to determine conformational equilibria in saccharides. In this subsection, we will demonstrate the relationships between a specific conformational descriptor, i.e., the torsional angle ω, describing the rotation of the hydroxymethyl group and the chemical shifts of atoms located in the vicinity of the rotating group. Furthermore, we will illustrate how these relationships can be used to determine the populations of the staggered rotamers *gt*, *gg*, and *tg* of this exocyclic group.

There is a consensus in the literature regarding the conformational variability of the monosaccharides β-d-Glc*p*-OMe, α-d-Man*p*-OMe, and α-d-Gal*p*-OMe (herein also referred for brevity as Glc, Man, and Gal, respectively), expressed through the relative populations of staggered rotamers *gt*:*gg*:*tg*. Both Glc and Man exhibit the trend *gt* ∼ *gg* > *tg*, whereas for Gal, due to the steric effect of the axially positioned hydroxyl group at C4, the analogous trend is *gt* > *tg* > *gg*. In both cases, the least populated conformer may be virtually absent, and neither the presence of a methyl glycoside at the anomeric position nor the type of anomeric configuration have a significant impact on the conformational preferences of the hydroxymethyl group. A compilation of experimental data and selected simulation results for the aforementioned three monosaccharides was presented in the work by Hansen et al. (Table 8 therein);^57^ other MD simulation results reported in conjunction with the rotamer distributions from experimental NMR data presented in Table 2 herein also reflect the expected trends.^52,99,100^

**Table 2:** Populations of the Three Staggered Conformers of the Hydroxymethyl Group in Methyl Hexopyranosides Expressed as the *gt*:*gg*:*tg* ratio and Determined from MD Simulations or on the Basis of Experimental ^3^ *J*_HH_ NMR Data

monosaccharide	temperature (K)	CHARMM	GROMOS	GLYCAM	NMR
β-d-Glc*p*-OMe	298	59.5:36:4.5	61:36:4	47:51:2	55:36:9^a^
α-d-Man*p*-OMe	310	59.5:35.5:5	65:31:4	38.5:59:2.5	55:36:9^b^
α-d-Gal*p*-OMe	310	53:2:45	59:10:31	75:9:16	68:3:29^c^
β-d-Glc*p*-OMe	343	56:37:7	59:36:5	51:45:4	
α-d-Man*p*-OMe	343	56:37:7	61:33:6	39.5:56.5:4	
α-d-Gal*p*-OMe	343	49:5:46	55:11.5:33.5	70:12:18	

a Populations based on ^3^ *J*_H5,H6_ from Ruda et al.^99^

b Olsson et al.^100^

c Dorst et al.^52^

Table 2 in this article contains data describing the *gt*:*gg*:*tg* ratio for Glc, Man, and Gal monosaccharides obtained from MD simulations conducted within three different carbohydrate-dedicated force fields (CHARMM, GROMOS, and GLYCAM), compared with the results of an analysis based on *J* coupling constants. While the previously identified trends in rotamer populations are preserved, there are quantitative differences between the predictions made by using different force fields (up to 26.5%) as well as between theoretical predictions and experimental data (up to 23%).

In contrast to approaches that obtain hydroxymethyl or ring-puckering thermodynamics directly from MD simulations for a given force field, the analysis described in the subsequent subsection uses MD mainly as a source of conformational sampling to compute conformation-dependent NMR chemical shifts, which are then combined with QM and experimental NMR data in an overdetermined optimization scheme to derive force-field-independent conformer populations. This strategy is designed to reduce the strong model dependence inherent to direct MD-based population estimates and to assess the robustness and transferability of chemical-shift-driven conformational analysis across different computational descriptions.

#### Relation between Chemical Shifts and ω Torsional Angle

We proceed to examine potential relationships between the fundamental descriptor of hydroxymethyl group conformation (i.e., the torsional angle ω) and the NMR chemical shift values of C and H atoms lying near or directly forming the rotating group. These atoms are C4, C5, C6, H5, H6*R*, and H6*S*. Figures 8 and 9 contain a graphical illustration of the corresponding relationships, obtained based on QM calculations for a large data set of molecular configurations (167–179 structures for each of the studied compounds). The QM data do not correspond to a typical QM energy scan procedure, which involves iteratively varying a single degree of freedom of the system and subsequent optimization of the geometry, but rather uses structures generated during MD simulations within a classical force field. This results in better sampling of degrees of freedom orthogonal to the torsional angle ω (particularly the conformations of hydroxyl and methoxyl groups) and provides a better representation of the configurational space explored by a real molecular system.

**Figure 8:**
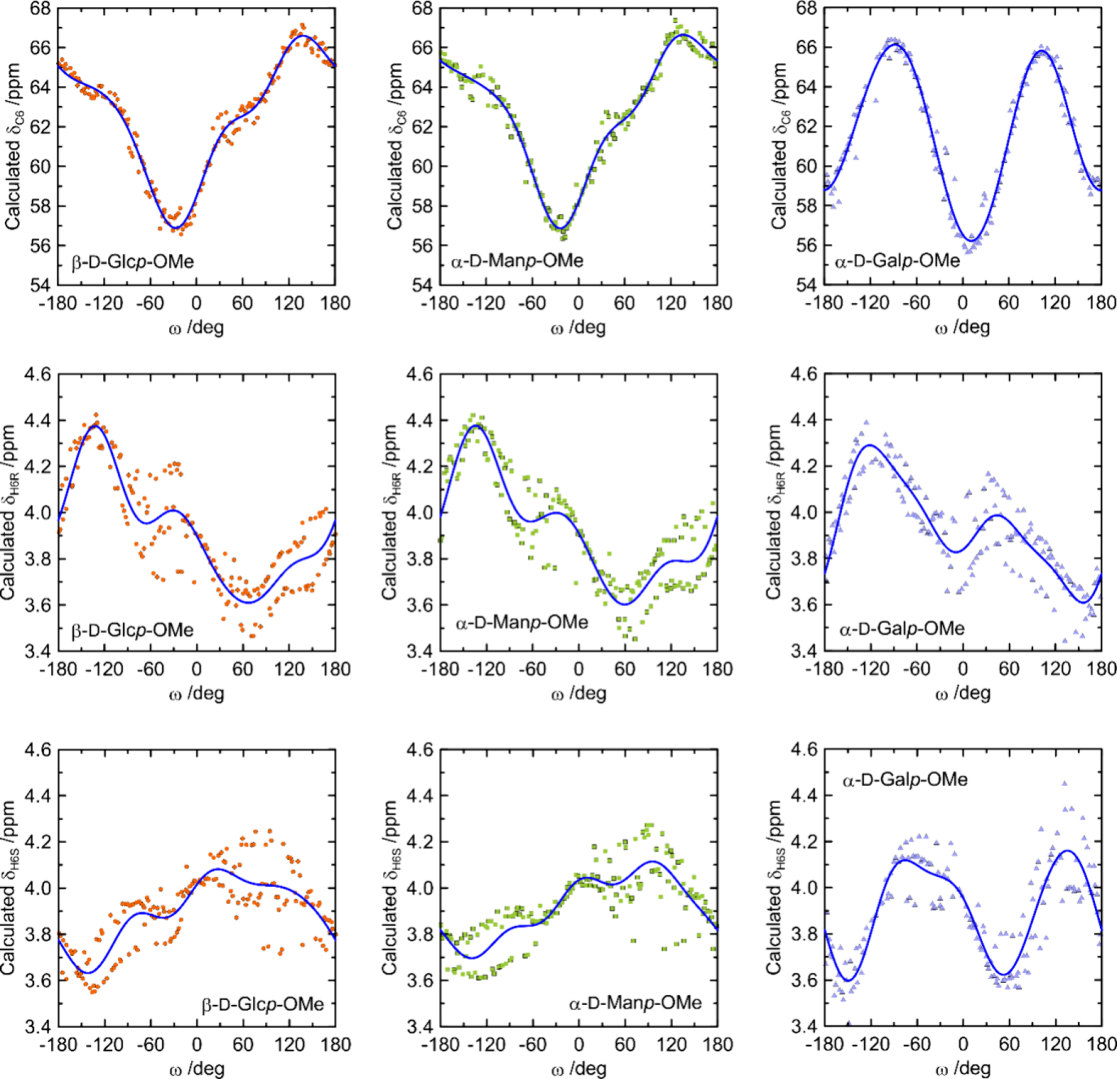
Calculated values of NMR chemical shifts δ_C6_, δ_H6R_ and δ_H6S_ plotted as a function of the ω torsion angle. The data points are fitted by eq 3 using coefficients from Table 3. The data correspond to the QM calculations using the MD-extracted structures and carried out at the DFT/ωB97XD/6-31+G­(d,p) level of theory.

**Figure 9:**
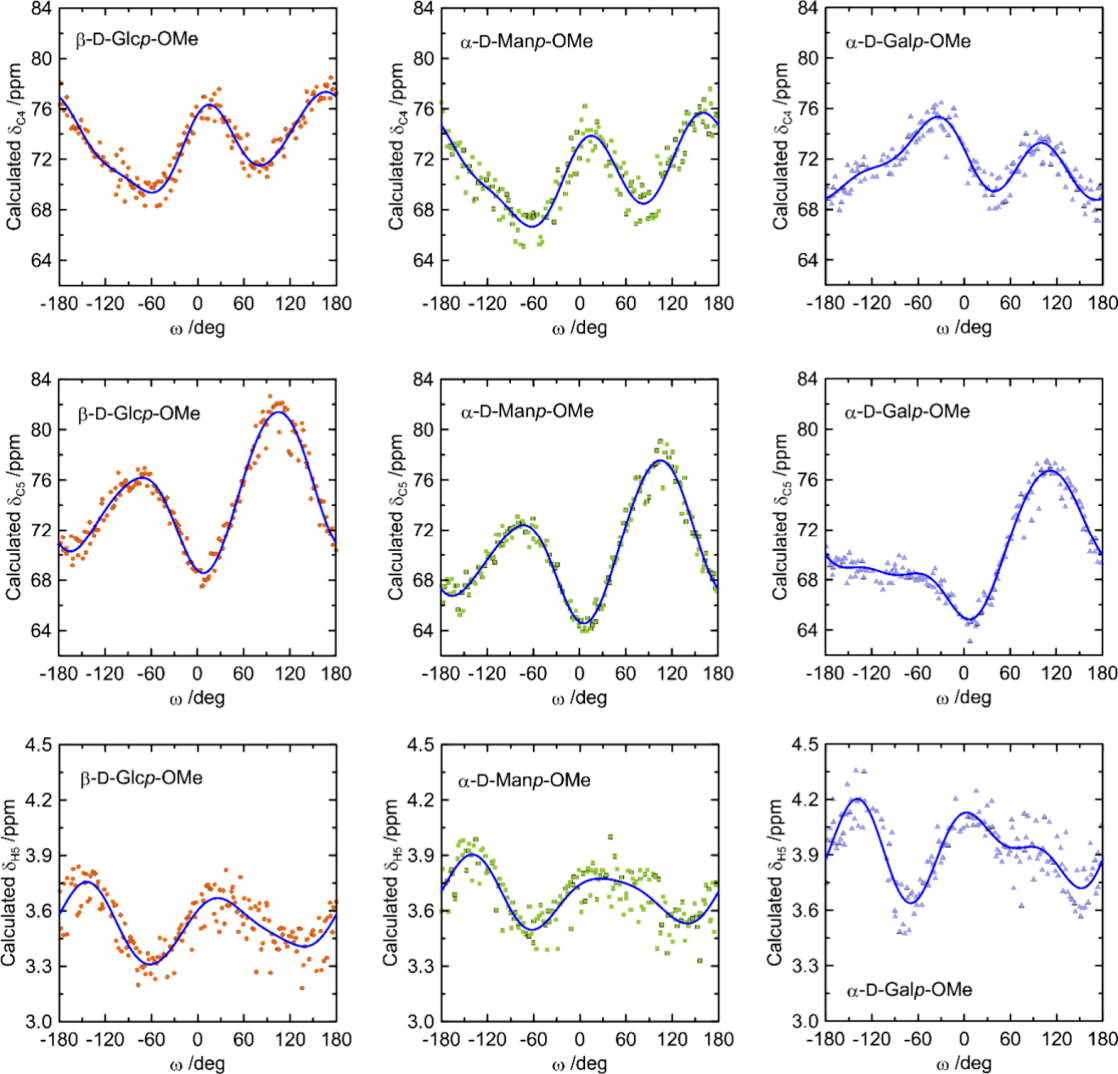
Calculated values of NMR chemical shifts δ_C4_, δ_C5_ and δ_H5_ plotted as a function of the ω torsion angle. The data points are fitted by eq 3. Other details are in Figure 8.

Regardless of the type of atom considered, the associated chemical shifts are strongly dependent on the value of the ω torsion angle. Each of the considered relationships can be approximated using eq 3, with nine coefficients determined based on minimal deviation from the QM data. The fitted coefficients are provided in Table 3. For hydrogen atoms, the deviations of values predicted by eq 3 range between: MAE = 0.0077–0.0111 ppm, whereas for carbon atoms, MAE = 0.18–1.31 ppm. When considering these MAE values relative to the total range of chemical shift variations, it is evident that chemical shifts associated with carbon atoms exhibit smaller deviations from eq 3 predictions and thus a lower dependence on conformational descriptors other than ω, compared to those of hydrogen atoms.

**Table 3:** Coefficients of eq 3, Used to Fit the ω vs. δ_H_ and ω vs. δ_C_ Data for Three Methyl Hexopyranosides^a^

monosaccharide	atom	α_0_	α_1_	α_2_	α_3_	α_4_	β_1_	β_2_	β_3_	β_4_
β-d-Glc*p*-OMe	C6	62.464	3.939	–0.744	–0.858	–0.227	3.595	0.731	1.866	0.963
	H6*R*	3.923	0.286	–0.071	0.098	0.030	1.967	2.161	0.676	9.196
	H6*S*	3.900	–0.176	0.039	–0.053	0.018	2.161	2.105	1.601	–0.527
	C4	73.345	1.934	2.634	–1.022	–0.336	0.967	–0.119	1.994	–3.508
	C5	74.353	–2.397	–4.641	0.366	–0.318	1.093	5.825	1.527	–0.162
	H5	3.527	–0.027	–0.171	0.045	–0.011	0.516	1.983	0.670	1.089
α-d-Man*p*-OMe	C6	62.631	4.014	–0.873	–0.771	–0.294	3.513	0.466	1.786	1.292
	H6*R*	3.928	0.284	–0.067	0.109	–0.017	1.992	2.091	0.746	0.425
	H6*S*	3.920	–0.1755	0.042	–0.024	0.027	2.087	2.082	–3.471	–0.050
	C4	70.997	–2.057	2.846	–1.322	0.150	0.851	–0.055	2.056	0.706
	C5	70.461	–2.490	–4.604	0.345	–0.298	1.053	5.855	1.833	0.308
	H5	3.682	–0.016	–0.155	0.053	0.002	–0.468	1.835	0.893	1.939
α-d-Gal*p*-OMe	C6	61.741	1.502	–4.156	0.393	–0.103	2.641	6.050	1.056	1.132
	H6*R*	3.936	0.088	–0.189	0.025	–0.039	0.896	0.918	0.590	0.498
	H6*S*	3.904	0.046	0.239	0.088	0.028	1.262	1.760	–0.896	0.431
	C4	71.654	1.645	1.663	1.296	0.053	0.462	2.127	1.190	0.778
	C5	70.047	–4.007	2.718	–0.585	0.318	0.868	2.557	1.412	–3.481
	H5	3.927	–0.026	–0.175	0.136	–0.004	–0.467	2.033	0.731	0.775

a The corresponding data and fitting functions are illustrated in Figures 8 and 9. The units of *α* and β coefficients are [ppm] and [rad], respectively.

The parameter fitting for eq 3 was performed separately for each of the considered atoms and compounds, resulting in a total of 18 individual equations. The very similar nature of the dependencies for C6, H6*R*, and H6*S* in Glc and Man suggests the possibility of reducing the number of equations and, in the context of system characterization, indicates a minor influence of the orientation of the C2 hydroxyl group on chemical shifts within the hydroxymethyl group. The opposite situation occurs for Gal, which in every case exhibits a significantly different course of the δ_C_(ω) and δ_H_(ω) dependencies compared to Glc and Man; this is intuitively consistent with expectations based on the more pronounced effect of the orientation of the hydroxyl group at C4 on the conformation of hydroxymethyl group. Furthermore, while the variability in the relationships for Glc and Man concerning C4, C5, and H5 is similar, non-negligible differences exist in the values of the α_0_ coefficient, resulting in relative shifts in the corresponding δ_C_(ω) and δ_H_(ω) values on the *y*-axis (Figure 9) for these two compounds.

It is worth noting that the α_0_ parameter in eq 3 is coupled with the σ_ref_ parameter from eq 2, and a change in σ_ref_ by any value results in an identical change in the α_0_ parameter. This is important to emphasize because the relationships presented in Figures 8 and 9 were obtained using additional modifications to the σ_ref_ parameter compared to the common values reported in the previous subsection. Specifically, an additional shift in the σ_ref_ value was applied, equal to

–1.407 ppm (common value for all compounds and C6 atoms);
–0.050 ppm (common value for all compounds and H6*R* and H6*S* atoms);
- 0.330 ppm (Glc, C4 and C5 atoms);
- 0.105 ppm (Glc, H5 atom);
- 0.527 ppm (Man, C4 and C5 atoms);
–0.067 ppm (Man, H5 atom);
- 0.692 ppm (Gal, C4 and C5 atoms);
–0.028 ppm (Gal, H5 atom).

These values are intended to improve the agreement between experimental data and QM data by minimizing deviations in the predicted values in relation to the originally applied scheme (relying on the selection of the σ_ref_ value as common for all compounds and all atoms of a given type). This additional refinement is based on an analogous minimization of deviations while considering only a subset of the data set, as specified above. Such an approach is necessary in the context of using NMR chemical shifts in conformational analysis, which will be explained in a subsequent subsection. The data presented in Figures 8 and 9, as well as the coefficients in Table 3, take into account the presence of these additional modifications to the σ_ref_ values.

#### Using MD Simulations to Predict NMR Chemical Shifts

In the first stage of this study aimed at interpreting the conformational variability of the hydroxymethyl group in the context of NMR chemical shift values, we used eq 3 with the appropriate coefficients from Table 3 to calculate the average chemical shift values of atoms C4, C5, C6, H5, H6*R*, and H6*S* using trajectories from MD simulations carried out using three force fields. This procedure is analogous to applying the Karplus equation and its numerous variations, aiming to obtain the average value of a given NMR parameter (here: a set of chemical shifts) for a given set of conformational descriptor values (here: a set of ω torsional angle values). The results are presented in Tables 4 and 5, with their graphical representation in Figure 10.

**Table 4:** Average Values of NMR Chemical Shifts δ_C4_, δ_C5_ and δ_C6_ Calculated from MD Simulations for Three Different Force Fields by Using eq 3 and Coefficients from Table 3 ^a^

monosaccharide^b^	temperature (K)	atom	CHARMM	GROMOS	GLYCAM	experiment^c^
Glc	343	C4	71.40	71.58	71.00	70.69
Man	343	C4	68.56	68.94	67.82	67.79
Gal	343	C4	69.63	69.78	70.45	70.19
Glc	343	C5	75.78	75.31	75.89	76.78
Man	343	C5	71.79	71.14	71.72	73.45
Gal	343	C5	70.79	70.11	70.97	71.54
Glc	298	C6	61.69	61.47	61.09	61.54
Man	310	C6	61.92	61.70	61.26	61.75
Gal	310	C6	61.01	60.84	62.03	62.01
Glc	343	C6	61.76	61.50	61.34	61.82
Man	343	C6	61.95	61.73	61.36	61.92
Gal	343	C6	61.04	60.94	62.04	62.06

a The graphical illustration of these data is shown in Figure 10.

b Glc, Man, and Gal are used for brevity to represent β-d-Glc*p*-OMe, α-d-Man*p*-OMe, and α-d-Gal*p*-OMe, respectively.

c ^13^C NMR chemical shifts at 343 K from Jansson et al.^92^

**Table 5:** Average Values of NMR Chemical Shifts δ_H5_, δ_H6*R*_ and δ_H6*S*_ Calculated from MD Simulations for Three Different Force Fields by Using eq 3 and Coefficients from Table 3 ^a^

monosaccharide^b^	temperature (K)	atom	CHARMM	GROMOS	GLYCAM	experiment^c^
Glc	343	H5	3.37	3.39	3.35	3.46
Man	343	H5	3.71	3.73	3.67	3.61
Gal	343	H5	3.91	3.90	3.92	3.89
Glc	343	H6*R*	3.96	3.97	3.96	3.92
		H6*S*	3.77	3.77	3.79	3.74
Man	343	H6*R*	3.96	3.97	3.93	3.90
		H6*S*	3.77	3.76	3.83	3.78
Gal	343	H6*R*	3.80	3.82	3.79	3.76
		H6*S*	3.83	3.87	3.90	3.76

a The graphical illustration of these data is shown in Figure 10.

b Glc, Man, and Gal are used for brevity to represent β-d-Glc*p*-OMe, α-d-Man*p*-OMe, and α-d-Gal*p*-OMe, respectively.

c ^1^H NMR chemical shifts at 343 K from Jansson et al.^92^

**Figure 10:**
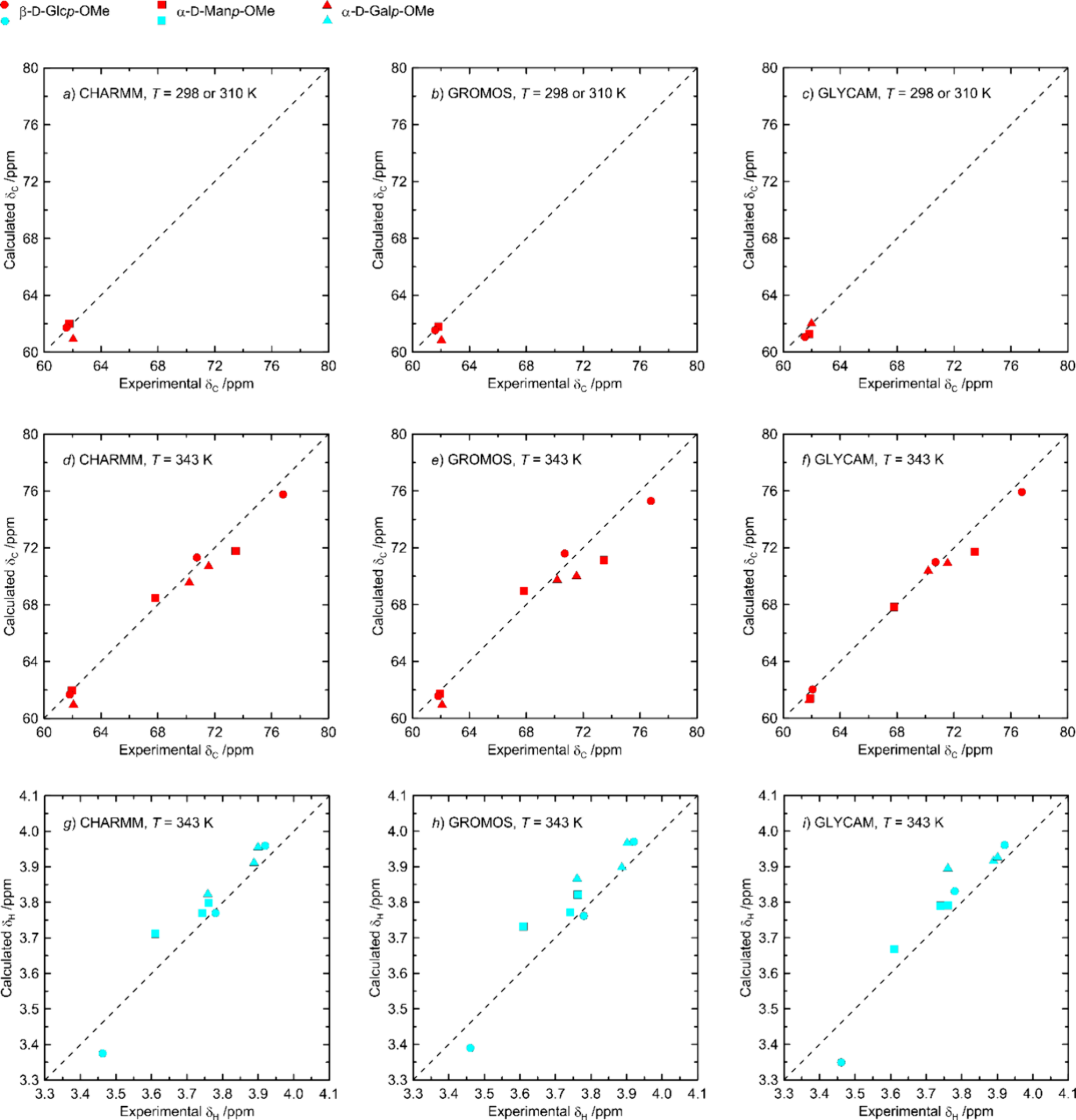
Average values of chemical shifts δ_C4_, δ_C5_, δ_C6_, δ_H5_, δ_H6R_ and δ_H6S_ calculated for the three considered monosaccharides from the MD simulations within three different force fields by using eq 3 and coefficients from Table 3.

Despite the relatively clear reproduction of the trend in chemical shift variability for data corresponding to the higher temperature (343 K), the applied procedure reveals several deviations (MAE ranging from 0.32 to 1.03 ppm for C atoms and 0.051 to 0.060 ppm for H atoms; see Table S6). Furthermore, for the δ_C6_ values measured at lower temperatures (298 and 310 K), the experimental trend is not reproduced in two out of three MD simulation sets (CHARMM and GROMOS force fields). Paradoxically, for this latter subset of data, the deviation from the experiment is very small in each case (MAE = 0.32–0.44 ppm), and a partial reason for the failure to capture the trend, aside from the inherent limitations of the method and models used in the MD simulations, is the very small range of δ_C6_ variability among experimental data for considered compounds (0.24 ppm for 343 K and 0.47 for 298/310 K). The expected trend, however, is correctly reproduced in simulations using the GLYCAM force field.

The most accurate predictions of δ_C_ values (MAE of 0.32 or 0.54 ppm, depending on the temperature and set of chemical shifts) are offered by the GLYCAM force field, followed by CHARMM and GROMOS. In the case of δ_H_, CHARMM provides the best accuracy (MAE = 0.051 ppm), although GROMOS and GLYCAM perform only slightly worse. It is also worth noting that, compared to the other two force fields, GLYCAM predicts a noticeably different distribution of *gt*:*gg*:*tg* populations (see Table 2). In contrast, when using the *J* coupling-derived and theoretical *gt*:*gg*:*tg* ratios to evaluate agreement of theory vs experiment, the GROMOS and CHARMM force fields offer much better predictions than GLYCAM (16% vs 16% vs. 30.5% per data point per compound, respectively; see Table 2 for *gt*:*gg*:*tg* populations).

Based on the values in Table 4, it is possible to examine the temperature dependence of δ_C6_, which has also been estimated experimentally. The relevant comparison is provided in Table 6. It can be observed that while the trend of variability was correctly reproduced in all cases, only GLYCAM, which most accurately predicts C6 chemical shift values, is able to capture the qualitative difference between Glc and Man vs Gal, specifically the significantly weaker temperature dependence of δ_C6_ observed in the case of Gal.

**Table 6:** Temperature-Dependence NMR Chemical Shifts of C6 for β-d-Glc*p*-OMe, α-d-Man*p*-OMe, and α-d-Gal*p*-OMe, Presented as Δ*δ*_C6_ (ppb·K^–1^)­^a^

monosaccharide	CHARMM	GROMOS	GLYCAM	experiment^b^
Glc	1.56	0.67	5.56	6.22
Man	0.91	0.91	3.03	5.15
Gal	0.91	3.03	0.30	1.52

a Data retrieved from values collected in Table 4.

b ^13^C NMR chemical shifts at 343 K used to calculate the temperature-dependence were obtained from Jansson et al.^92^

#### Conformational Preferences of the Hydroxymethyl Group Predicted Based on NMR Chemical Shifts

The approach described in the previous subsection is strictly based on the model (force field) used for MD simulations. As shown in Table 2, there are non-negligible differences between the models, leading to varying accuracy in the predicted chemical shift values (Tables 4 and 5, Figure 10). A completely different approach is to determine conformational preferences based on known experimental chemical shift values and knowledge of the chemical shifts corresponding to specific rotamers. This approach is equivalent to using a set of *J* coupling constants corresponding to several conformers of a given molecule to predict the populations of those conformers that best match the experimental data. This subsection presents this method in the context of chemical shifts and the monosaccharides Glc, Man, and Gal.

The first essential step is estimating the conformation-dependent values of the considered chemical shifts (δ_C6_, δ_H6*R*_, δ_H6*S*_, δ_C4_, δ_C5_, and δ_H5_) corresponding to individual rotamers of the hydroxymethyl group (*gt*, *gg*, and *tg*). This was done using eq 3 and the coefficients from Table 3 for subsets of the full range of torsional angle ω values corresponding exclusively to a given rotamer. The data are presented in Tables 7 and 8. Additionally, QM-derived values averaged using eq 1 and obtained via the procedure used to calculate the full set of chemical shifts are provided. In the latter case, as the calculations relied on a set of optimized structures rather than a thermodynamically representative MD trajectory, the effect of temperature is not explicitly accounted for. These data were also modified using the appropriate σ_ref_ values, as described in previous subsection.

**Table 7:** Average Values of Conformation-Dependent NMR Chemical Shifts δ_C4,_ δ_C5_, and δ_C6_ Corresponding to Selected Types of Staggered Conformers of the Hydroxymethyl Group^a^

	CHARMM			GROMOS			GLYCAM			QM^b^		
monosaccharide @ temperature	*gt*	*gg*	*tg*	*gt*	*gg*	*tg*	*gt*	*gg*	*tg*	*gt*	*gg*	*tg*
atom C4												
Glc (298 K)	72.41	69.58	77.12	72.81	69.70	76.75	72.37	69.58	76.82	72.16	69.01	77.32
Man (310 K)	69.73	66.90	75.20	70.21	67.07	74.90	69.69	66.91	75.01	69.57	66.88	74.88
Gal (310 K)	71.19	74.15	68.97	70.49	73.79	69.16	71.14	74.05	69.08	70.99	74.46	68.79
Glc (343 K)	72.44	69.61	76.97	72.83	69.74	76.71	72.42	69.62	76.79			
Man (343 K)	69.76	66.93	75.16	70.23	67.13	74.85	69.72	66.95	74.97			
Gal (343 K)	71.20	74.13	68.99	70.54	73.76	69.20	71.15	74.04	69.11			
C5												
Glc (298 K)	76.69	75.87	73.07	75.90	75.57	73.30	76.88	75.80	73.59	76.64	75.92	72.15
Man (310 K)	72.72	72.05	69.36	71.90	71.66	69.60	72.92	71.98	70.01	72.86	71.97	68.68
Gal (310 K)	72.46	68.38	70.69	70.71	68.35	71.67	72.33	68.37	71.30	72.00	68.34	69.87
Glc (343 K)	76.68	75.84	73.07	75.93	75.52	73.29	76.86	75.75	73.52			
Man (343 K)	72.71	72.02	69.40	71.92	71.60	69.50	72.90	71.94	69.89			
Gal (343 K)	72.47	68.37	70.73	70.75	68.34	71.73	72.33	68.35	71.35			
C6												
Glc (298 K)	62.68	59.54	65.68	62.56	59.19	65.64	62.72	59.37	65.74	62.50	59.62	65.44
Man (310 K)	62.54	60.31	65.84	62.38	59.79	65.82	62.61	60.18	65.93	62.71	59.62	65.44
Gal (310 K)	62.43	64.14	59.18	60.85	64.51	59.72	62.31	64.26	59.50	63.00	64.49	59.09
Glc (343 K)	62.69	59.55	65.66	62.57	59.18	65.62	62.73	59.38	65.72			
Man (343 K)	62.55	60.31	65.84	62.39	59.79	65.79	62.62	60.17	65.91			
Gal (343 K)	62.43	64.13	59.22	60.88	64.49	59.77	62.30	64.22	59.55			

a Calculations involved only selected fragments of the MD data, representing a given conformer; eq 3 and coefficients from Table 3 were used. In the case of QM data, calculations involved 50 structures per one conformer and the data were averaged using eq 1.

b Temperature-independent.

**Table 8:** Average Values of Conformation-Dependent NMR Chemical Shifts δ_H5_, δ_H6*R*_, and δ_H6*S*_ Corresponding to Selected Types of Staggered Conformers of the Hydroxymethyl Group^a^

	CHARMM			GROMOS			GLYCAM			QM^b^		
monosaccharide @ temperature	*gt*	*gg*	*tg*	*gt*	*gg*	*tg*	*gt*	*gg*	*tg*	*gt*	*gg*	*tg*
H5												
Glc (298 K)	3.58	3.33	3.50	3.59	3.34	3.52	3.58	3.33	3.50	3.57	3.31	3.56
Man (310 K)	3.74	3.51	3.62	3.74	3.52	3.63	3.73	3.51	3.61	3.71	3.48	3.63
Gal (310 K)	3.94	3.69	3.84	3.96	3.69	3.79	3.94	3.69	3.81	3.90	3.66	3.92
Glc (343 K)	3.58	3.33	3.51	3.59	3.34	3.52	3.58	3.33	3.50			
Man (343 K)	3.74	3.51	3.62	3.74	3.52	3.63	3.73	3.51	3.62			
Gal (343 K)	3.94	3.70	3.84	3.96	3.70	3.79	3.94	3.69	3.81			
H6*R*												
Glc (298 K)	4.03	3.88	3.84	4.04	3.88	3.84	4.03	3.88	3.85	4.05	3.86	3.80
Man (310 K)	4.05	3.84	3.88	4.04	3.85	3.88	4.05	3.84	3.89	4.21	3.86	3.80
Gal (310 K)	3.69	4.09	3.88	3.67	4.10	3.96	3.69	4.09	3.93	3.83	4.08	3.85
Glc (343 K)	4.03	3.88	3.84	4.04	3.88	3.84	4.03	3.88	3.85			
Man (343 K)	4.05	3.84	3.88	4.04	3.85	3.87	4.05	3.84	3.88			
Gal (343 K)	3.69	4.09	3.88	3.68	4.09	3.96	3.69	4.09	3.93			
H6*S*												
Glc (298 K)	3.62	3.97	3.89	3.63	3.97	3.90	3.62	3.97	3.88	3.54	3.93	3.92
Man (310 K)	3.62	3.97	3.88	3.63	3.98	3.90	3.62	3.97	3.87	3.54	3.92	3.92
Gal (310 K)	3.93	4.04	3.71	3.95	4.07	3.67	3.93	4.05	3.68	3.83	4.04	3.74
Glc (343 K)	3.62	3.97	3.89	3.63	3.97	3.91	3.63	3.97	3.89			
Man (343 K)	3.62	3.97	3.88	3.63	3.98	3.91	3.63	3.98	3.88			
Gal (343 K)	3.93	4.04	3.71	3.95	4.07	3.67	3.93	4.05	3.69			

a Calculations involved only selected fragments of the MD data, representing a given conformer. Other details are in Table 7.

b Temperature-independent.

As can be seen, the conformation-dependent chemical shift values depend very little on the force field or temperature. The QM data are also similar to those predicted by the individual force fields. This provides a perspective for eliminating differences in force field predictions collected in Table 2, as well as the associated limitations in predicting the final *gt*:*gg*:*tg* ratio.

The final step in the procedure was to optimize the *gt*:*gg*:*tg* populations using eqs 4 and 5. Since the optimization can include the full data set (all experimental δ_C6_, δ_H6*R*_, δ_H6*S*_, δ_C4_, δ_C5_, and δ_H5_ values and their corresponding conformation-dependent values for the *gt*, *gg*, and *tg* rotamers from Tables 7 and 8) or its subsets, we performed three independent optimizations covering:

- the full data set;
- only δ_C6_, δ_H6*R*_, and δ_H6*S*_;
- only δ_C4_, δ_C5_, and δ_H5_.

The optimized *gt*, *gg*, and *tg* populations are summarized in Table 9 and illustrated in Figures 11, S4, and S5. Of course, other combinations of data selection are possible (not tested here), as well as limiting the number of data types, to just two chemical shifts, which would lead to a determined system of equations (three variables corresponding to *gt*, *gg*, and *tg* populations, two constraints for two chemical shift values, and the condition that the sum of the populations equals 100%). We examined also this case, but it did not yield meaningful results, which indicates that a better approach is to use a larger number of chemical shift types and optimize conformer populations within an overdetermined system of equations.

**Table 9:** Populations of the Three Staggered Conformers of a Hydroxymethyl Group Expressed as the *gt*:*gg*:*tg* Ratio and Determined by Using eqs 4 and 5 Combined with Conformation-Dependent NMR Chemical Shifts δ_C6_, δ_H6*R*_, δ_H6*S*_, δ_C4_, δ_C5_, and δ_H5_ Collected in Tables 7 and 8 ^a^

monosaccharide	CHARMM	GLYCAM	GROMOS	QM	NMR
	set of data: δ_C6_, δ_H6*R*_ and δ_H6*S*_				
Glc	65:31:4	64:31:5	67:27:6	48:38:14	55:36:9^b^
Man	50:34:16	52:40:8	55:33:12	37:48:15	55:36:9^c^
Gal	63:0:37	69:0:31	71:0:29	76:0:24	68:3:29^d^
	set of data: δ_C4_, δ_C5_ and δ_H5_				
Glc	54:46:0	54:46:0	50:50:0	59:41:0	55:36:9^b^
Man	42:58:0	44:56:0	40:60:0	55:45:0	55:36:9^c^
Gal	53:3:45	59:0:41	61:7:32	78:0:22	68:3:29^d^
	set of data: δ_C6_, δ_H6*R*_, δ_H6*S*_, δ_C4_, δ_C5_ and δ_H5_				
Glc	60:40:0	61:39:0	61:39:0	48:40:12	55:36:9^b^
Man	54:46:0	56:44:0	57:43:0	38:40:22	55:36:9^c^
Gal	63:0:37	71:0:29	71:0:29	69:0:31	68:3:29^d^

a The experimentally derived rotamer populations of the *ω* torsion angle serving as a reference in eqs 4 and 5 are given as well.

b Populations based on ^3^ *J*_H5,H6_ from Ruda et al.^99^

c Olsson et al.^100^

d Dorst et al.^52^

**Figure 11:**
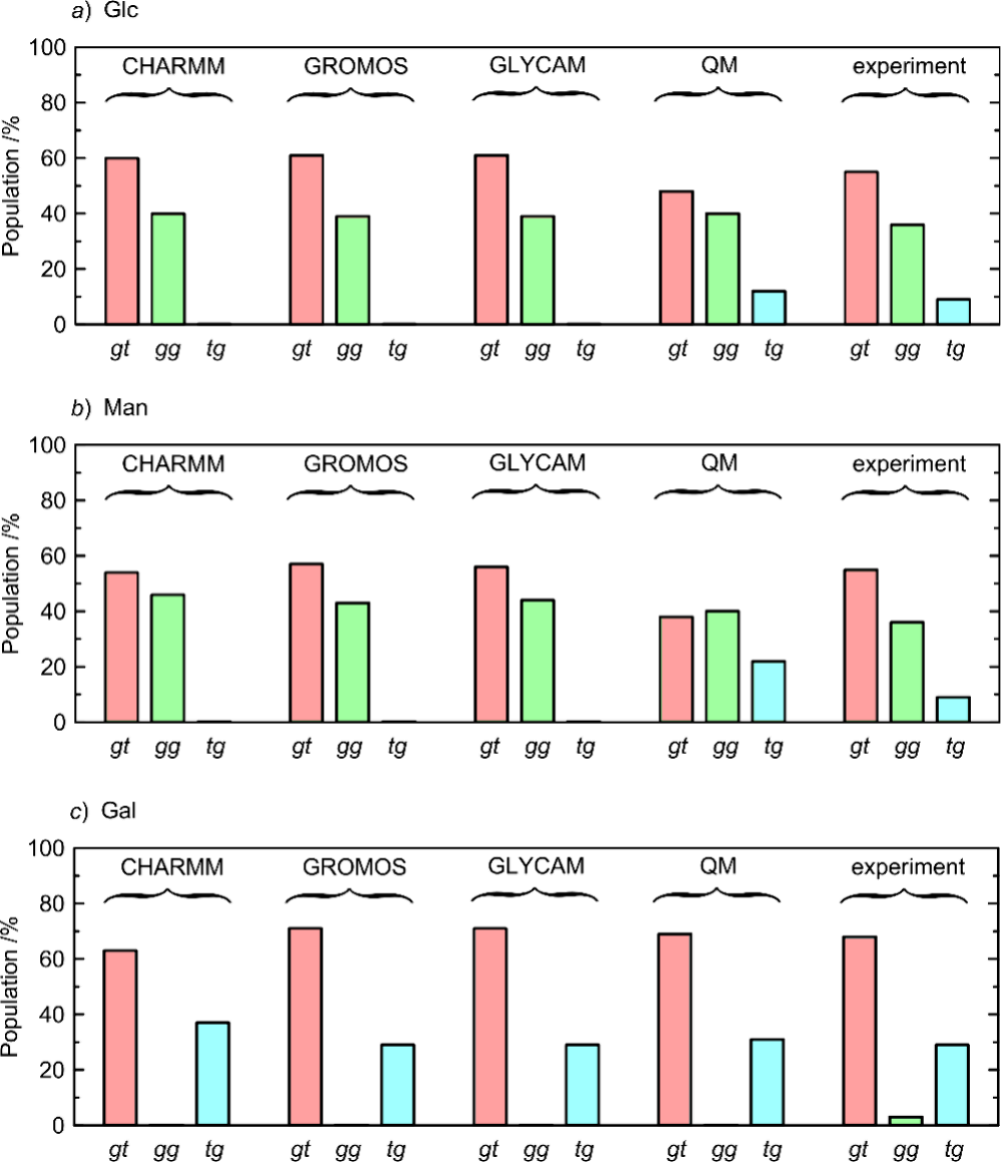
Populations of the three staggered conformers of the hydroxymethyl group (*gt*,*gg* and *tg*) in (a) Glc, (b) Man and (c) Gal determined by using eqs 4 and 5 combined with conformation-dependent NMR chemical shifts δ_C4_, δ_C5_, δ_H5_ δ_C6_, δ_H6*R*_ and δ_H6*S*_ collected in Tables 7 and 8. The ‘experiment’ label denotes estimations relying on the *J* coupling constants.

Regardless of the input data — whether corresponding to different force fields or QM data — the predicted populations of staggered rotamers match trends known from the literature: *gt* ∼ *gg* > *tg* for Glc and Man and *gt* > *tg* > *gg* for Gal. Within a given compound and reference data set, the largest deviations from the rest of the data are observed for results based on QM data. This is likely due to the neglect of off-equilibrium structures, i.e., those not corresponding to any (local or global) energy minimum, which is related to the geometry optimization procedure performed before calculating NMR parameters. A consequence of this is the relatively larger differences in conformation-dependent chemical shift values from Tables 7 and 8, compared to the corresponding values obtained from MD simulations. However, the differences between populations determined using MD simulations within different force fields are rather small, reaching at most 8%.

Much larger differences are observed in the results obtained based on different reference data sets, i.e., the full set of chemical shifts (Figure 11) vs two of its subsets (Figures S4 and S5). The largest deviation from the *J* coupling constant-based populations corresponds to populations estimated using the C4, C5, and H5 chemical shift subset (MAE = 6.2–10.6% with an average of 9.0%), followed by populations derived from the full data set (MAE = 4.7–6% with an average of 5.3%) and those from the C6, H6*R*, and H6*S* subset (MAE = 3.6–7.3% with an average of 5.1%). The differences between the last two cases are very small. However, from the perspective of minimizing potential inaccuracies associated with values of chemical shift of specific type, using the widest possible data set is recommended.

#### Potential Difficulties and Limitations in Using NMR Chemical Shifts to Determine Molecular Conformation

Determining the universal value of σ_ref_, which depends on the available data set of chemical shifts for atoms of a given type and, at the same type, describes accurately every individual chemical shift is challenging. The independently and directly determined chemical shifts for the reference compound (dioxane) are associated with excessive inaccuracy. In practice, the most accurate σ_ref_ value appears to be the one obtained by minimizing deviations between the calculated and experimental values across the entire data set (separately for C and H atoms). However, when higher accuracy is required — such as when using chemical shifts to determine conformational preferences — a similar procedure should involve only subsets of chemical shift values corresponding to the specific atoms considered in such an analysis. This leads to additional modifications in the values of σ_ref_. The necessity of using different σ_ref_ values for different groups of atoms is one of the most significant shortcomings of the approach discussed in previous subsection.

As demonstrated in the case of the conformational preferences of the hydroxymethyl group, the final average chemical shift values determined from MD trajectories are sensitive to the applied model and, in this specific case, to the predicted populations of *gt*:*gg*:*tg*. This sensitivity explains difficulties such as obtaining a consistent trend in the variability of δ_C6_ for the three monosaccharides: even relatively small changes in the *gt*:*gg*:*tg* population (Table 2) can lead to qualitative changes in this trend. Therefore, even minor inaccuracies in the model used for MD simulations can result in significant deviations in the output data.

Thus, the most reasonable approach appears to be using conformation-dependent chemical shift values determined for a discrete set of well-defined conformers and optimizing the final populations using eq 4 or its equivalent.

## Conclusions

The characterization of the structure and conformational properties of saccharides in aqueous solution relies largely on NMR spectroscopy and the interpretation of relevant observables, such as chemical shifts and *J* coupling constants. In this article, we explore the relationship between chemical shift values and the conformation of monosaccharide molecules using a combination of standard MD simulations and QM calculations referenced against NMR experimental data.

The procedure for obtaining NMR parameters corresponding to experimental chemical shift data based on averaging the QM-calculation results relying on MD simulation-extracted data using eq 1 accurately reflects the variability of measured chemical shifts for ^1^H (δ_H_) and ^13^C (δ_C_) within a group of 11 monosaccharide entities studied in this work. Additionally, with the exception of the *O*-methyl group, where some systematic deviations occur, the results of calculations also capture the variability of topologically analogous atoms within this group of monosaccharides. In light of these results, the proposed calculation scheme could potentially be useful for the qualitative identification of compounds, as the calculation results are sensitive to seemingly small structural differences that are not related to conformational variability.

Both δ_C_ and δ_H_ depend on the molecular conformation of the monosaccharide, although this dependence may not be as evident as in the case of, for example, *J* coupling constants. The determined dependencies corresponding to specific conformer types can be used to estimate conformational equilibria, as demonstrated here for: (1) the β-d-Ara*p*-OMe ring interconversion, and (2) the population of staggered rotamers of hydroxymethyl group of β-d-Glc*p*-OMe, α-d-Man*p*-OMe, and α-d-Gal*p*-OMe.

In the first of these cases adjusting the ^1^ *C*_4_:^4^ *C*_1_ ratio based on either δ_H_ or δ_C_ values, analogous to the two-state model, clearly indicates the prevalence of the ^1^ *C*_4_ inverted chair conformer, in line with independent estimations based on ^3^ *J*_HH_, ^3^ *J*_CH_, ^2^ *J*_HH_ and ^1^ *J*_CH_ coupling constants as well as with results of enhanced-sampled MD simulations.

In the case of the rotating hydroxymethyl group, dependencies of δ_C_ and δ_H_ on the torsion angle ω were determined for C4, C5, C6, H5, H6*R*, and H6*S* atoms of β-d-Glc*p*-OMe, α-d-Man*p*-OMe, and α-d-Gal*p*-OMe. These dependencies are expressed as a mathematical function given in eq 3, with empirical coefficients varying from one compound to the other. The resulting mathematical relationships can be used to transform the structural data from MD simulations, specifically, the torsion angle ω, into ensemble-averaged values of δ_C_ and δ_H_. This approach is analogous to the use of Karplus-type equations in that it relates the torsion angle ω to NMR observables, but instead of coupling constants, it provides δ_C_ and δ_H_ values for the atoms surrounding the rotatable C–C bond. However, this procedure is strongly dependent on the quality of the model applied in MD simulations. As demonstrated here, even extensively validated saccharide-dedicated force fields differ in their predictions and such differences result in non-negligible variations in predicted chemical shifts.

An alternative method for data analysis, partially mitigating the inaccuracies inherent to force fields, involves using conformation-dependent chemical shifts δ_C_ and δ_H_, corresponding to selected types of molecular conformations. This procedure, applied to the conformers of the hydroxymethyl group in β-d-Glc*p*-OMe, α-d-Man*p*-OMe, and α-d-Gal*p*-OMe, predicts the *gt*:*gg*:*tg* ratio to be in excellent agreement with the estimations based on *J* coupling constants.

The methodology developed herein linking the derived NMR chemical shifts to molecular conformation of the compound under investigation, which integrates data from MD simulations and QM calculations, is based on optimizing the populations of a few well-defined conformers and appears to be particularly well-suited for carbohydrates. Most of the key conformational degrees of freedom such as ring conformation and exocyclic group orientations can be accurately described using just a few (2–3) distinct conformers, to which the proposed procedure can be applied. We foresee that the methodology should be applicable to also glycosidic linkage conformation analysis.

To sum up, the careful analysis of chemical shifts relying on MD simulations and QM calculations can be a complementary method supporting other, standard analyses of NMR observables in determining the conformational properties of saccharides.

## Supplementary Material



## Data Availability

The computer programs, codes, and their versions used in the computational part of the study are listed in the Methods section. GROMACS topology files, Gaussian09 input files, molecular coordinates (including MD trajectories), and QM calculation results are available from the authors upon request. The remaining data supporting the findings of this paper are available within the paper and the Supporting Information.

## Notes

^1^H NMR spectrum of β-d-Ara*p*-OMe (Figure S1); 1DLR NMR spectrum of β*-* d-Ara*p*-OMe (Figure S2); calculated vs experimental NMR chemical shifts of β-d-Ara*p*-OMe (^4^ *C*_1_ conformer) (Figure S3); MAE of ^13^C and ^1^H NMR chemical shifts (experiment vs theory, compound- and atom-based analyses) (Tables S1 and S2); MAE of ^3^ *J*_HH_ coupling constants (experiment vs theory) (Table S3);^3^ *J*_HH_ data for α-d-Xyl*p* and β-d-Xyl*p*-OMe (Table S4); ring-distortion free energies (Table S5); MAE of δ_C6_ and δ_H6_ (experiment vs theory) (Table S6); and hydroxymethyl group populations derived from δ_C6_, δ_H6*R*_, δ_H6*S*_ (Figure S4) or from δ_C4_, δ_C5_, and δ_H5_ (Figure S5) (PDF)
