# Supplementary material for: Resolving Conformational Preferences of Monosaccharides from 1H and 13C NMR Chemical Shifts Using an Integrated MD and QM Approach
Source: J Chem Inf Model. 2026 Jul 6;66(14):8361–81. doi: 10.1021/acs.jcim.6c01310 (PMC13417880; doi:10.1021/acs.jcim.6c01310)
Supplement: Supplementary file 1 [file ci6c01310_si_001.pdf]

Resolving Conformational Preferences of Monosaccharides from ^{1}H and ^{13}C NMR Chemical Shifts Using an Integrated MD and QM Approach

Wojciech Plazinski[a,]* and Göran Widmalm[b,]*

[a] Jerzy Haber Institute of Catalysis and Surface Chemistry, Polish Academy of Sciences, 30-239 Krakow, Poland

[b] Department of Chemistry, Arrhenius Laboratory, Stockholm University, S-106 91 Stockholm, Sweden

*Correspondence: wojtek_plazinski@o2.pl or goran.widmalm@su.se

Supporting Information

Table of Contents

Figure S1	^{1}H NMR spectrum of β-D-Ara*p*-OMe	S2
Figure S2	1DLR NMR spectrum of β-D-Ara*p*-OMe	S3
Figure S3	Calculated *vs.* experimental NMR chemical shifts of β-D-Ara*p*-OMe, $^{4}C_{1}$	S4
Table S1	MAE ^{13}C and ^{1}H NMR chemical shifts experiment *vs.* theory; compounds	S4
Table S2	MAE ^{13}C and ^{1}H NMR chemical shifts experiment *vs.* theory; atom-based	S5
Table S3	MAE $^{3}J_{HH}$ experiment *vs.* theory; compounds	S5
Table S4	$^{3}J_{HH}$ of D-Xyl*p* and β-D-Xyl*p*-OMe	S5
Table S5	Ring-distortion free energies; compounds	S6
Table S6	MAE δ_{C6} and δ_{H6} experiment *vs.* theory	S6
Figure S4	Hydroxymethyl group populations from δ_{C6}, δ_{H6R} and δ_{H6S}; compounds	S7
Figure S5	Hydroxymethyl group populations from δ_{C4}, δ_{C5} and δ_{H5}; compounds	S8

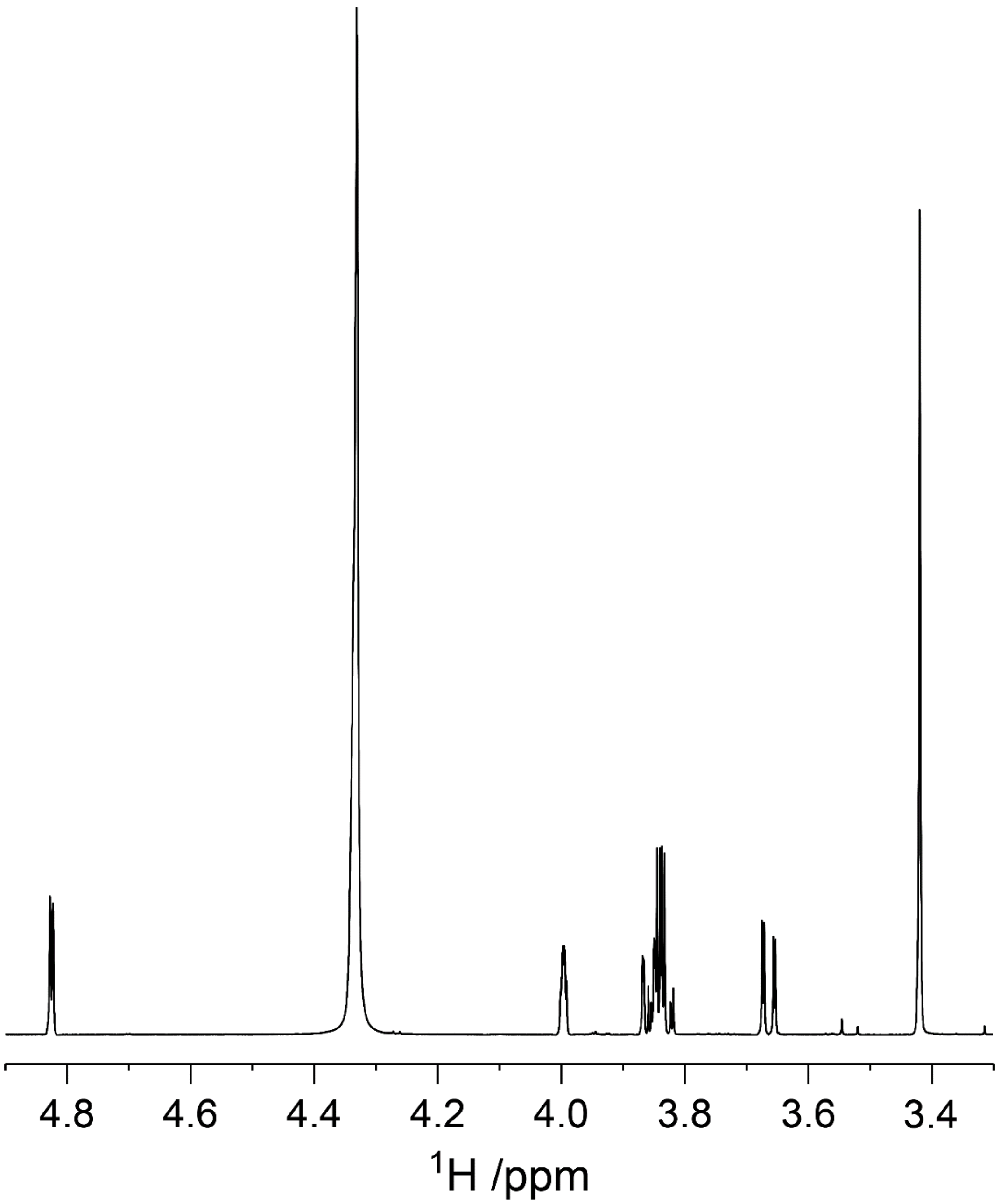

Figure S1.^{1}H NMR spectrum at 700 MHz of β-D-Ara*p*-OMe in D_2O at 343 K.

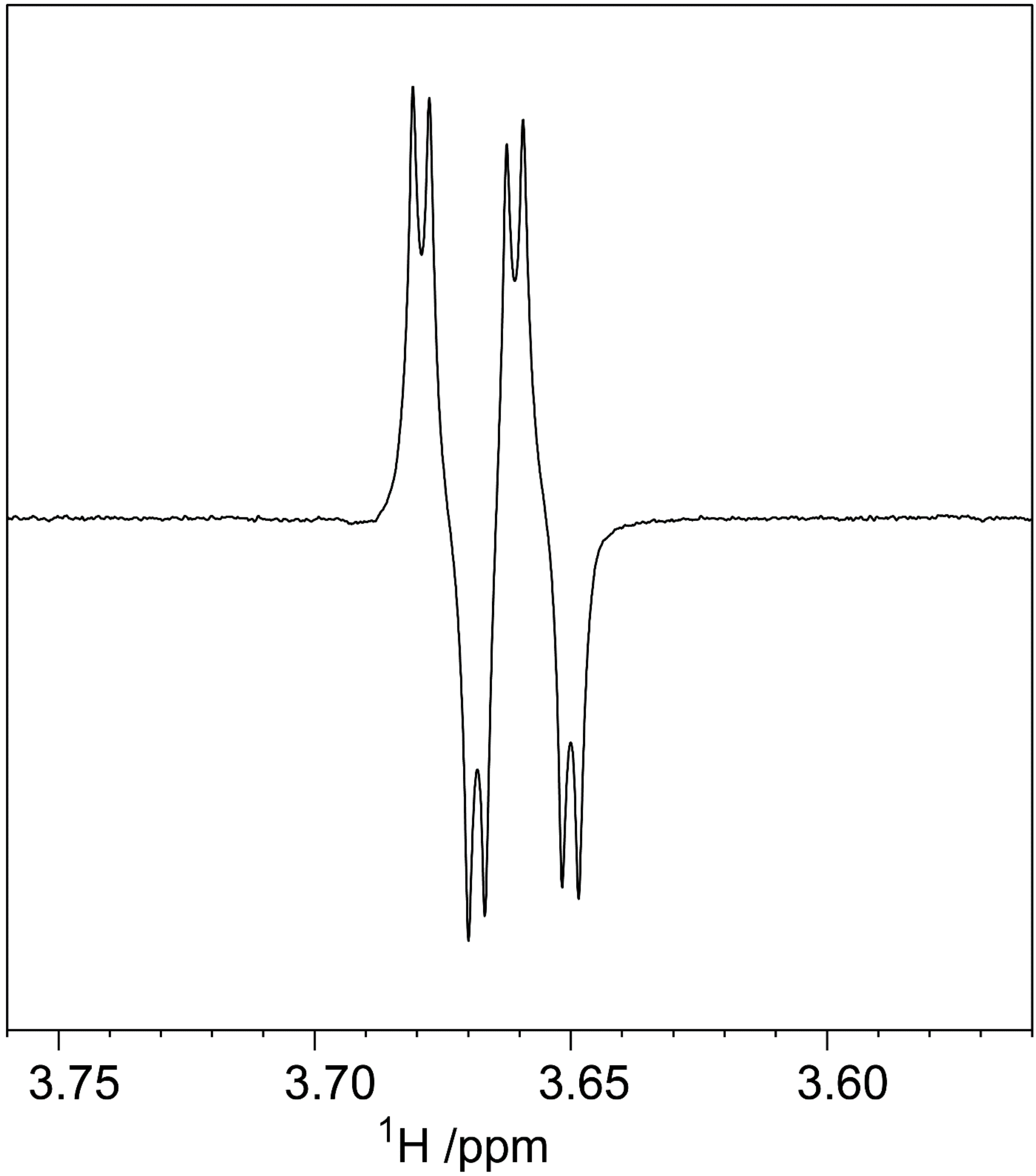

Figure S2. The H5$_{pro\text{-}S}$ resonance of β-D-Ara*p*-OMe form a 1DLR NMR experiment at 700 MHz and 343 K with selective excitation at the anomeric carbon C1; the $^{3}J_{C1,H5pro\text{-}S}$ coupling constant is obtained as the anti-phase peak separation.

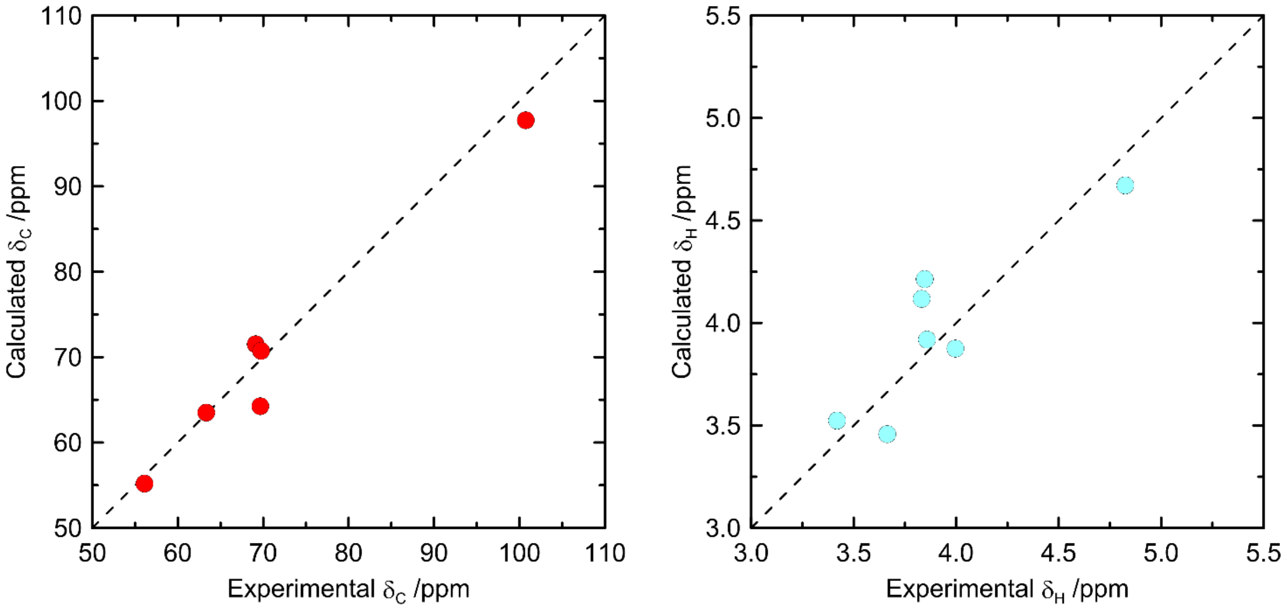

Figure S3. NMR chemical shifts δ_C (left panel) and δ_H (right panel) calculated for β-D-Ara*p*-OMe being in the $^4C_1$ conformation and averaged by using eqn (1) compared to the experimental data. The theoretical data correspond to the QM calculations using the MD-extracted structures and carried out at the DFT/ωB97XD/6-31+G(d,p) level of theory. The data for β-D-Ara*p*-OMe corresponding to the $^1C_4$ chair conformation of the sugar ring are shown in Figures 4 and 5 of the main manuscript.

Table S1. Mean absolute errors (MAE) calculated for experimental *vs.* theoretical values of δ_C and δ_H for all studied compounds. The data correspond to Figures 4 and 5 of the main manuscript.

Compound	MAE for δ_C [ppm]	MAE for δ_H [ppm]
α-L-Rha*p*-OMe	0.82	0.028
β-L-Rha*p*-OMe	1.04	0.088
α-L-Fuc*p*-OMe	1.18	0.041
β-L-Fuc*p*-OMe	1.10	0.098
α-D-Xyl*p*	0.52	0.052
β-D-Xyl*p*	0.55	0.045
β- D-Xyl*p*-OMe	1.19	0.060
β-D-Ara*p*-OMe ($^1C_4$)	1.22	0.079
β-D-Ara*p*-OMe ($^4C_1$)	2.15	0.186
β-D-Glc*p*-OMe	0.87	0.077
α-D-Man*p*-OMe	1.19	0.064
α-D-Gal*p*-OMe	0.93	0.089
All [a]	0.96	0.066

[a] for β-D-Ara*p*-OMe in the $^1C_4$ conformation.

Table S2. Mean absolute errors (MAE) calculated for experimental *vs.* theoretical values of δ_C and δ_H for all topologically-equivalent atoms of the studied compounds. The data correspond to Figure 6 of the main manuscript.

Atom	MAE for δ_C [ppm]	Atom	MAE for δ_H [ppm]
C1	1.89	H1	0.079
C2	0.76	H2	0.043
C3	0.45	H3	0.058
C4	0.48	H4	0.056
C5	0.96	H5	0.094
C6	0.48	H6	0.064
OMe	2.22	OMe [a]	0.077

[a] value averaged over three protons.

Table S3. Mean absolute errors (MAE) calculated for experimental *vs.* theoretical values of $^3J_{HH}$ for α-D-Xyl*p*, β-D-Xyl*p*, β-D-Xyl*p*-OMe and β-D-Ara*p*-OMe. The data correspond to Figure 7 of the main manuscript and Figure S3.

Compound	MAE for $^3J_{HH}$ [Hz]
α-D-Xyl*p*	0.34
β-D-Xyl*p*	0.35
β-D-Xyl*p*-OMe	0.26
β-D-Ara*p*-OMe ($^1C_4$)	0.51
β-D-Ara*p*-OMe ($^4C_1$)	3.77
All [a]	0.36

[a] for β-D-Ara*p*-OMe in the $^1C_4$ conformation.

Table S4. Scalar three-bond coupling constants in hertz from ^{1}H NMR spectra of D-xylopyranose and methyl D-xylopyranoside in D_2O at 343 K, compared to the theoretical predictions from MD simulations data combined with a Haasnoot-Altona equation.

$^3J_{HH}$	α-D-Xyl*p*		β-D-Xyl*p*		β-D-Xyl*p*-OMe	
	Expt.	Pred.	Expt.	Pred.	Expt.	Pred.
H1,H2	3.67	3.16	7.89	8.59	7.77	8.29
H2,H3	9.44	9.51	9.33	9.19	9.27	9.23
H3,H4	8.86	9.21	9.09	8.93	8.95	8.91
H4,$H5_{pro\text{-}R}$	5.54	4.83	5.58	4.85	5.47	4.84
H4,$H5_{pro\text{-}S}$	10.50	10.54	10.54	10.56	10.33	10.38

Table S5. The ring-distortion free energies (ΔF) calculated using metadynamics simulations for selected compounds. Two types of conformational transitions were considered: ring inversion ($^4C_1 \rightarrow {}^1C_4$) and ring distortion from the regular chair to boat/skew (*B/S*) conformers ($^4C_1 \rightarrow B/S$).

Compound	ΔF ($^4C_1 \rightarrow {}^1C_4$) [kJ/mol]	ΔF ($^4C_1 \rightarrow B/S$) [kJ/mol]
α-D-Xyl*p*	10.9	23.1
β-D-Xyl*p*	16.4	14.2
β-D-Xyl*p*-OMe	15.8	13.9
β-D-Ara*p*-OMe	−9.2	16.3

Table S6. The mean absolute error (MAE) values calculated for experimental *vs.* theoretical values of δ_{C6} and δ_{H6} for β-D-Glc*p*-OMe, α-D-Man*p*-OMe and α-D-Gal*p*-OMe. The data correspond to Figure 10 of the main manuscript.

Atom	Estimation of conformer populations	MAE for δ [ppm]
C6	MD within CHARMM, 298 or 310 K	0.44
	MD within GROMOS, 298 or 310 K	0.43
	MD within GLYCAM, 298 or 310 K	0.32
C4, C5, C6	MD within CHARMM, 343 K	0.73
	MD within GROMOS, 343 K	1.03
	MD within GLYCAM, 343 K	0.54
H5, H6*R*, H6S	MD within CHARMM, 343 K	0.051
	MD within GROMOS, 343 K	0.060
	MD within GLYCAM, 343 K	0.060

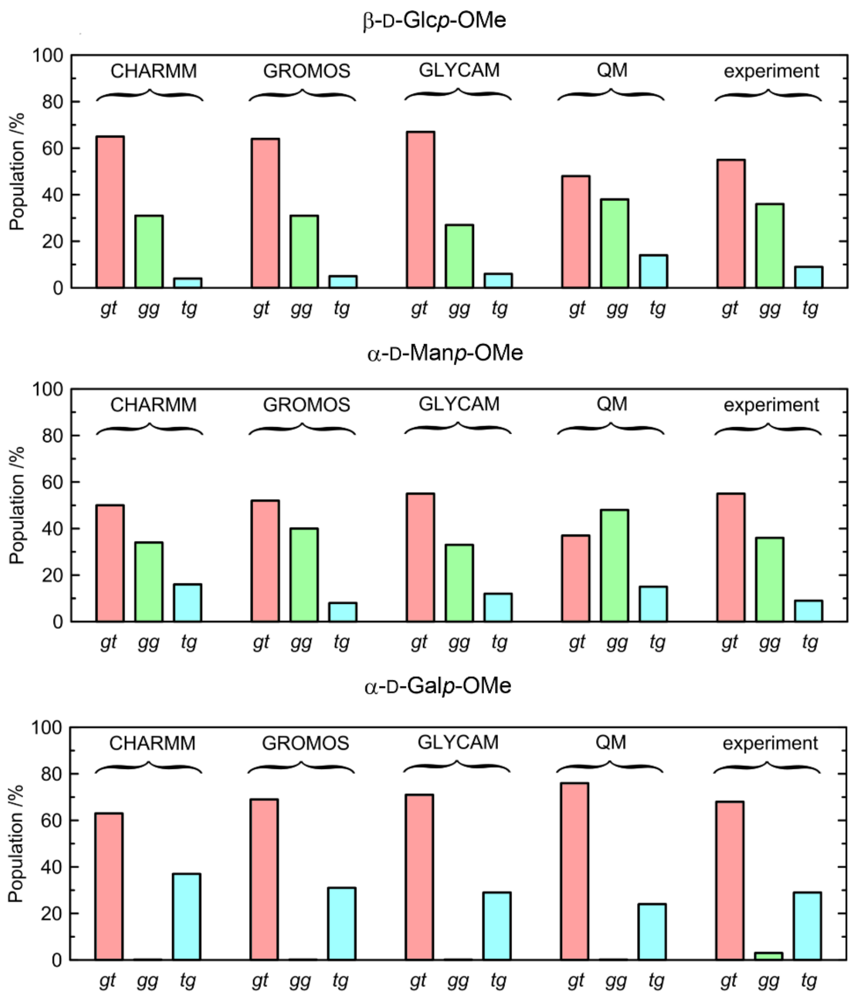

Figure S4. Populations of the three staggered conformers of hydroxymethyl group (*gt*, *gg* and *tg*) determined by using eqns (4) and (5) combined with conformation-dependent δ_{C6}, δ_{H6R} and δ_{H6S} collected in Tables 7 and 8. The 'experiment' label denotes estimates relying on the *J* coupling constants.

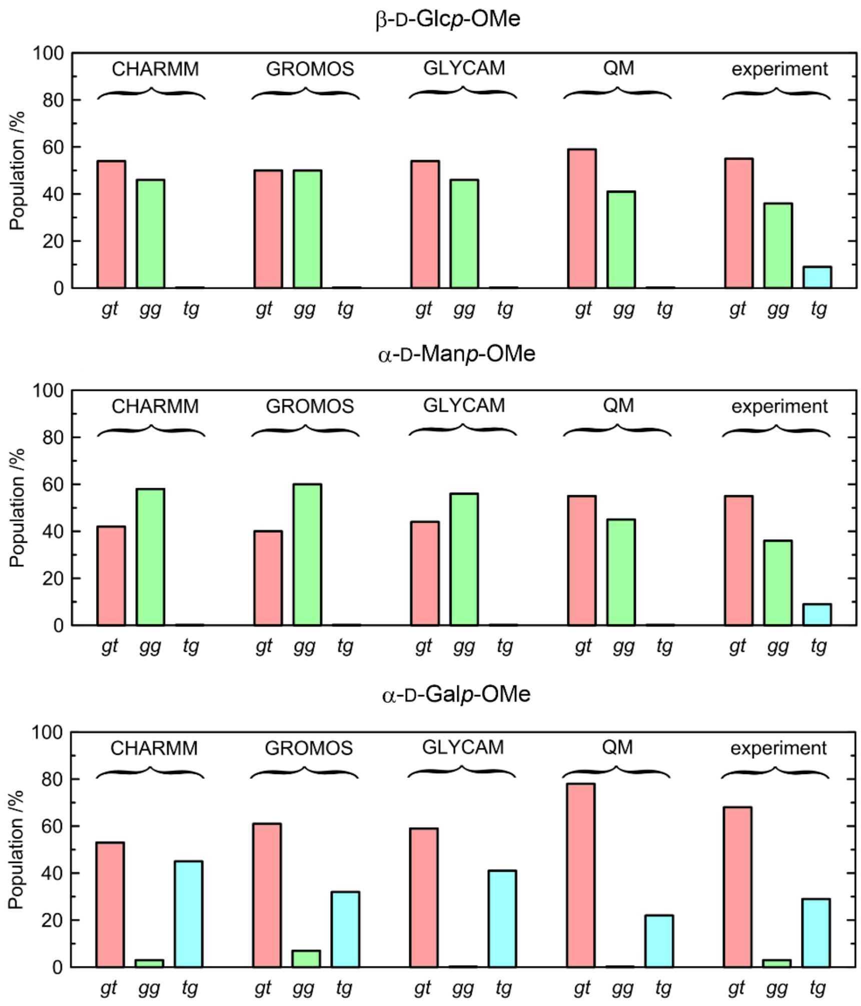

Figure S5. Populations of the three staggered conformers of hydroxymethyl group (*gt*, *gg* and *tg*) determined by using eqns (4) and (5) combined with conformation-dependent δ_{C4}, δ_{C5} and δ_{H5} collected in Tables 7 and 8. The 'experiment' label denotes estimates relying on the *J* coupling constants.
